# Transancestral fine-mapping of four type 2 diabetes susceptibility loci highlights potential causal regulatory mechanisms

**DOI:** 10.1093/hmg/ddw048

**Published:** 2016-02-23

**Authors:** Momoko Horikoshi, Lorenzo Pasquali, Steven Wiltshire, Jeroen R. Huyghe, Anubha Mahajan, Jennifer L. Asimit, Teresa Ferreira, Adam E. Locke, Neil R. Robertson, Xu Wang, Xueling Sim, Hayato Fujita, Kazuo Hara, Robin Young, Weihua Zhang, Sungkyoung Choi, Han Chen, Ismeet Kaur, Fumihiko Takeuchi, Pierre Fontanillas, Dorothée Thuillier, Loic Yengo, Jennifer E. Below, Claudia H.T. Tam, Ying Wu, Gonçalo Abecasis, David Altshuler, Graeme I. Bell, John Blangero, Noél P. Burtt, Ravindranath Duggirala, Jose C. Florez, Craig L. Hanis, Mark Seielstad, Gil Atzmon, Juliana C.N. Chan, Ronald C.W. Ma, Philippe Froguel, James G. Wilson, Dwaipayan Bharadwaj, Josee Dupuis, James B. Meigs, Yoon Shin Cho, Taesung Park, Jaspal S. Kooner, John C. Chambers, Danish Saleheen, Takashi Kadowaki, E. Shyong Tai, Karen L. Mohlke, Nancy J. Cox, Jorge Ferrer, Eleftheria Zeggini, Norihiro Kato, Yik Ying Teo, Michael Boehnke, Mark I. McCarthy, Andrew P. Morris

**Affiliations:** ^1^Wellcome Trust Centre for Human Genetics, Nuffield Department of Medicine, University of Oxford, Oxford, UK,; ^2^Oxford Centre for Diabetes, Endocrinology and Metabolism, Radcliffe Department of Medicine, University of Oxford, Oxford, UK,; ^3^Program of Predictive and Personalized Medicine of Cancer (PMPPC), Germans Trias i Pujol University Hospital and Research Institute, Badalona, Spain,; ^4^Josep Carreras Leukaemia Research Institute, Badalona, Spain,; ^5^CIBER de Diabetes y Enfermedades Metabólicas Asociadas (CIBERDEM), Barcelona, Spain,; ^6^Department of Biostatistics and Center for Statistical Genetics, University of Michigan, Ann Arbor, MI, USA,; ^7^Department of Human Genetics, Wellcome Trust Sanger Institute, Hinxton, Cambridgeshire, UK,; ^8^Saw Swee Hock School of Public Health,; ^9^Department of Medicine, Yong Loo Lin School of Medicine, National University of Singapore, National University Health System, Singapore, Singapore,; ^10^Department of Diabetes and Endocrinology, JR Tokyo General Hospital, Tokyo, Japan,; ^11^Department of Diabetes and Metabolic Diseases, Graduate School of Medicine and; ^12^Department of Integrated Molecular Science on Metabolic Diseases, 22nd Century Medical and Research Center, The University of Tokyo, Tokyo, Japan,; ^13^Department of Public Health and Primary Care, Institute of Public Health, University of Cambridge, Cambridge, UK,; ^14^Department of Cardiology, Ealing Hospital NHS Trust, Southall, Middlesex, UK,; ^15^Department of Epidemiology and Biostatistics,; ^16^Department of Genomics of Common Disease, School of Public Health,; ^17^National Heart and Lung Institute, Cardiovascular Sciences, Hammersmith Campus,; ^18^Imperial College Healthcare NHS Trust, and; ^19^Department of Medicine, Imperial College London, London, UK,; ^20^Interdisciplinary Program in Bioinformatics and; ^21^Department of Statistics, Seoul National University, Seoul, Republic of Korea,; ^22^Department of Biostatistics, Harvard School of Public Health, Boston, MA, USA,; ^23^Department of Biostatistics, Boston University School of Public Health, Boston, MA, USA,; ^24^Genomics and Molecular Medicine, CSIR-Institute of Genomics & Integrative Biology, New Delhi, India,; ^25^Department of Gene Diagnostics and Therapeutics, Research Institute, National Center for Global Health and Medicine, Tokyo, Japan,; ^26^Program in Medical and Population Genetics, Broad Institute, Cambridge, MA, USA,; ^27^Integrative Genomics and Modelization of Metabolic Diseases CNRS UMR8199, Lille Institute of Biology, E.G.I.D – FR3508 European Genomics Institute of Diabetes, Lille, France,; ^28^Human Genetics Center, School of Public Health, University of Texas Health Science Center at Houston, Houston, TX, USA,; ^29^Department of Medicine and Therapeutics,; ^30^Hong Kong Institute of Diabetes and Obesity, and; ^31^Li Ka Shing Institute of Health Sciences, Chinese University of Hong Kong, Hong Kong, China,; ^32^Department of Genetics, University of North Carolina, Chapel Hill, NC, USA,; ^33^Department of Biology, Massachusetts Institute of Technology, Cambridge, MA, USA,; ^34^Department of Genetics and; ^35^Department of Medicine, Harvard Medical School, Boston, MA, USA,; ^36^Department of Molecular Biology,; ^37^Diabetes Research Center (Diabetes Unit), Department of Medicine,; ^38^Center for Human Genetic Research, Department of Medicine, and; ^39^General Medicine Division, Massachusetts General Hospital, Boston, MA, USA,; ^40^Departments of Medicine and Human Genetics, University of Chicago, Chicago, IL, USA,; ^41^Department of Genetics, Texas Biomedical Research Institute, Houston, TX, USA,; ^42^Blood Systems Research Institute, San Francisco, CA, USA,; ^43^Department of Laboratory Medicine and Institute for Human Genetics, University of California, San Francisco, San Francisco, CA, USA,; ^44^Department of Natural Science, University of Haifa, Haifa, Israel,; ^45^Departments of Medicine and Genetics, Albert Einstein College of Medicine, New York, USA,; ^46^Department of Physiology and Biophysics, University of Mississippi Medical Center, Jackson, MS, USA,; ^47^School of Biotechnology, Jawaharlal Nehru University, New Delhi, India,; ^48^National Heart, Lung, and Blood Institute's Framingham Heart Study, Framingham, MA, USA,; ^49^Department of Biomedical Science, Hallym University, Chuncheon, Republic of Korea,; ^50^Department of Biostatistics and Epidemiology, Center for Non-Communicable Diseases, University of Pennsylvania, Philadelphia, PA, USA,; ^51^Cardiovascular & Metabolic Disorders Program, Duke-NUS Graduate Medical School Singapore, Singapore,; ^52^School of Medicine, Vanderbilt University, Nashville, TN, USA,; ^53^Genomic Programming of Beta-cells Laboratory, Institut d'Investigacions August Pi i Sunyer (IDIBAPS), Barcelona, Spain,; ^54^Life Sciences Institute and; ^55^Department of Statistics and Applied Probability, National University of Singapore, Singapore,; ^56^Oxford NIHR Biomedical Research Centre, Oxford University Hospitals Trust, Oxford, UK and; ^57^Department of Biostatistics, University of Liverpool, Liverpool, UK

## Abstract

To gain insight into potential regulatory mechanisms through which the effects of variants at four established type 2 diabetes (T2D) susceptibility loci (*CDKAL1*, *CDKN2A-B*, *IGF2BP2* and *KCNQ1*) are mediated, we undertook transancestral fine-mapping in 22 086 cases and 42 539 controls of East Asian, European, South Asian, African American and Mexican American descent. Through high-density imputation and conditional analyses, we identified seven distinct association signals at these four loci, each with allelic effects on T2D susceptibility that were homogenous across ancestry groups. By leveraging differences in the structure of linkage disequilibrium between diverse populations, and increased sample size, we localised the variants most likely to drive each distinct association signal. We demonstrated that integration of these genetic fine-mapping data with genomic annotation can highlight potential causal regulatory elements in T2D-relevant tissues. These analyses provide insight into the mechanisms through which T2D association signals are mediated, and suggest future routes to understanding the biology of specific disease susceptibility loci.

## Introduction

Genome-wide association studies (GWAS) of type 2 diabetes (T2D) have been extremely successful in identifying loci contributing genetic effects to disease susceptibility in multiple ancestry groups (1–5). These loci are typically characterized by common variant association signals, defined by a lead single-nucleotide polymorphism (SNP) with minor allele frequency (MAF) of at least 5%, in the ancestry group in which it was discovered. The association signals often map to large genomic intervals because of extensive linkage disequilibrium (LD) between common variants within populations from the same ancestry group, making localization and identification of causal alleles at T2D susceptibility loci extremely challenging. Consequently, there has been limited progress in defining the molecular mechanisms through which the effects of GWAS loci on disease are mediated.

There is increasing evidence, however, that T2D association signals discovered in one ancestry group are transferrable across diverse populations (6–9). For the majority of established T2D susceptibility loci, common variant association signals are shared across ancestries. Furthermore, there is limited evidence across populations of heterogeneity in the allelic effects of lead SNPs identified through transancestral meta-analysis (10). This observation is consistent with a model in which the underlying causal variants are shared across ancestry groups, and thus arose prior to human population migration out of Africa. Under this assertion, we expect to enhance the fine-mapping resolution of causal alleles by combining GWAS across ancestry groups because of the increased sample size and as a result of differences in the structure of LD between diverse populations (11–13).

To harness the power of transancestral fine-mapping for localizing potential causal variants for T2D susceptibility, we have undertaken meta-analysis of GWAS in 22 086 cases and 42 539 controls from five ancestry groups: East Asian, European, South Asian, African American and Mexican American (Supplementary Material, Table S1). We focussed on four loci, mapping to/near *CDKAL1*, *CDKN2A-B*, *IGF2BP2* and *KCNQ1*, because they harbour the strongest signals of association across diverse ancestries, with no evidence of heterogeneity in allelic effects between populations (10). Previous ancestry-specific meta-analyses have reported lead SNPs attaining genome-wide significance (*P*< 5 × 10^−8^) at all four loci in European and East Asian descent populations (3,4), and at *KCNQ1*, also in African Americans (5). All four of the loci have a primary physiological impact on T2D susceptibility via β-cell dysfunction (4), and thus might be expected, a priori, to share similar mechanisms through which the GWAS signals are mediated.

Previous transancestral GWAS meta-analyses for T2D susceptibility (10) have been limited by imputation up to the relatively sparse reference panels from the International HapMap Consortium (14), which provides limited coverage of variation with MAF <5% across diverse populations. To improve fine-mapping resolution, we have undertaken imputation of each study up to the ‘all ancestries’ reference panel from the 1000 Genomes Project Consortium (15) (Phase 1 integrated release, March 2012) across the four loci. With these data, we aimed to: (i) statistically delineate distinct association signals arising from multiple causal variants in each locus through conditional analyses; (ii) re-evaluate the evidence for heterogeneity in allelic effects between ancestry groups for each distinct association signal; (iii) construct credible sets of variants that are most likely to drive each distinct association signal and thus most likely to incorporate causal alleles; and (iv) interrogate credible set variants for predicted functional annotation and regulatory sites in relevant tissues (primarily pancreatic islet β-cells) to provide insight into the potential causal mechanisms through which the effects of each distinct association signal on T2D susceptibility are mediated.

## Results

### Study overview

We considered a total of 18 studies, genotyped with a range of GWAS arrays, in 22 086 T2D cases and 42 539 controls (Supplementary Material, Table S1): seven of East Asian ancestry (9867 cases and 12 870 controls), five of European ancestry (4555 cases and 12 932 controls), four of South Asian ancestry (6196 cases and 13 775 controls), one of African American ancestry (631 cases and 2526 controls) and one of Mexican American ancestry (837 cases and 436 controls). At each of the four loci, the GWAS scaffold in each study was imputed up to the ‘all ancestries’ Phase 1 integrated reference panel (March 2012 release) from the 1000 Genomes Project Consortium (15) using IMPUTEv2 (16) or minimac (17). We excluded variants with MAF <1% from each study, after imputation, because our focus was on common and low-frequency association signals that are shared across diverse populations, and thus amenable to transancestral fine-mapping to improve localization of causal variants. We then retained ‘well-imputed’ variants, defined as attaining widely used thresholds (18) of IMPUTEv2 info ≥0.4 or minimac *r*^2^ ≥ 0.3, for downstream association analyses.

We began, in each study, by testing for association of T2D with each variant (MAF ≥1% and passing imputation quality control) across the four loci (Materials and Methods, Supplementary Material, Table S2). Variants passing quality control in <80% of the total sample size (i.e. in <51 700 individuals) were excluded from the transancestral meta-analysis. Our primary analysis combined association summary statistics across studies using MANTRA (19). This Bayesian method has been designed for transancestral meta-analysis and fine-mapping by allowing for heterogeneity in allelic odds ratios (ORs) between studies. Such heterogeneity can arise as a result of differential patterns of LD with a shared causal variant between diverse populations from distinct ancestry groups. However MANTRA can also allow for heterogeneity in allelic ORs arising from genuine effect size differences between ancestry groups, including the possibility of interaction with environmental risk factors that differ in exposure between diverse populations, or variable phenotype definition or ascertainment strategies across studies.

MANTRA incorporates a prior model of relatedness between studies to account for heterogeneity in allelic ORs, and has been demonstrated, by simulation, to improve detection and localization of causal variants compared with either a fixed- or random-effects transancestral meta-analysis (19,20). Here, the relatedness between studies has been developed by applying hierarchical clustering to the observed pair-wise differences in mean allele frequency across variants at the four loci, and highlighted three distinct ancestral clades (Supplementary Material, Fig. S1): (i) a single African American study (AfAm); (ii) a cluster of studies of East Asian ancestry (EAsia) and (iii) a cluster of studies of European, Mexican American and South Asian ancestry (Eur-MexAm-SAsia). The evidence in favour of association from MANTRA is measured by means of a Bayes' factor (BF). For completeness, we also combined association summary statistics across studies through traditional fixed-effects meta-analysis, which makes the limiting assumption of no heterogeneity in allelic ORs between studies (Materials and Methods).

### Identification of distinct association signals

There is increasing evidence of multiple ‘distinct’ association signals at established T2D susceptibility loci, each arising as a result of different causal variants acting independently or, *in cis*, on the same haplotype (4). The first stage in comprehensive fine-mapping of GWAS loci is thus to disentangle, statistically, these distinct association signals, and to localize the causal variants for each, in turn, on the basis of conditional analyses. In this framework, each distinct association signal can be represented by an ‘index variant’, here required to attain genome-wide significant evidence of association (MANTRA log_10_BF ≥6 and fixed-effects *P*< 5 × 10^−8^) in conditional transancestral meta-analysis (Materials and Methods). Across the four loci, we identified a total of seven distinct signals of association, three mapping to *KCNQ1*, two to *CDKN2A-B* and one each at *IGF2BP2* and *CDKAL1* (Table 1).

**Table 1.** Summary statistics from the conditional transancestral meta-analysis (22 086 cases and 42 539 controls) for distinct T2D association signals at each locus

Locus	Index SNP	Chr	Position (b37)	Alleles		Mean (range) *r*^2^ or info	MANTRA		Fixed-effects meta-analysis		
				Risk	Other		Log_10_BF	Log_10_BF heterogeneity	OR (95% CI)	*P*-value	Cochran's *Q P*-value
*IGF2BP2*	rs11705729	3	185 507 299	T	C	0.96 (0.74–1.00)	19.35	−0.05	1.14 (1.11–1.17)	1.3 × 10^−21^	0.49
*CDKAL1*	rs9368222	6	20 686 996	A	C	0.97 (0.74–1.00)	28.84	0.99	1.17 (1.14–1.21)	4.1 × 10^−30^	0.0058
*CDKN2A-B*	rs10965246	9	22 132 698	T	C	0.94 (0.79–1.00)	37.45	−0.03	1.31 (1.26–1.36)	8.4 × 10^−40^	0.0029
	rs10757282	9	22 133 984	C	T	0.92 (0.32–1.00)	10.31	0.01	1.12 (1.09–1.16)	2.0 × 10^−12^	0.17
*KCNQ1*	rs231353	11	2 709 019	G	A	0.93 (0.68–0.99)	9.29	−0.13	1.11 (1.07–1.14)	1.7 × 10^−11^	0.79
	rs233448	11	2 840 424	C	T	0.94 (0.84–1.00)	9.65	0.14	1.12 (1.09–1.16)	9.5 × 10^−12^	0.18
	rs2237897	11	2 858 546	C	T	0.75 (0.35–0.97)	9.79	0.17	1.19 (1.14–1.26)	7.7 × 10^−12^	0.35

Chr, chromosome; OR, odds ratio; CI, confidence interval.

The association of variants mapping to the *KCNQ1* locus with T2D susceptibility was initially established in GWAS of East Asian ancestry, and was localized to a <50 kb intronic region of the gene (21,22). Association of variants in this interval have been widely replicated, at genome-wide significance, across GWAS from multiple populations (3,4,5,10). However, the lead SNPs from East Asian and European ancestry meta-analyses are in only weak LD with each other (rs2237896 and rs163184, respectively; CEU *r*^2^ = 0.027, CHB + JPT *r*^2^ = 0.395). Meta-analyses of European ancestry GWAS (4,23) have also identified an additional association signal at this locus, ∼150 kb upstream of the recombination interval described above, and mapping to the *KCNQ1-OT1* transcript that controls regional imprinting (24). Through exact conditioning, our transancestral meta-analysis has formally demonstrated that the association at this locus can be delineated by three distinct signals (Supplementary Material, Fig. S2), two localized to the <50 kb *KCNQ1* intronic recombination interval (rs2237897, MANTRA log_10_BF = 9.79, *P*= 7.7 × 10^−12^; rs233448, MANTRA log_10_BF = 9.65, *P*= 9.5 × 10^−12^) and one mapping to *KCNQ1-OT1* (rs231353, MANTRA log_10_BF = 9.29, *P*= 1.7 × 10^−11^). After accounting for these three index variants in conditional analyses, no residual association signal attains genome-wide significance (maximum MANTRA log_10_BF = 3.38, *P*= 2.1 × 10^−5^, rs223448).

At the *CDKN2A-B* locus, association of T2D susceptibility with variants localized to a 12 kb intergenic recombination interval was first reported in GWAS of European descent (25), and then widely replicated across ancestry groups (3,4,5,10). Haplotype analyses have revealed that the association signal can best be explained by two partially correlated SNPs (rs10811661 and rs10757282, CEU *r*^2^ = 0.360) in the recombination interval, acting together, *in cis*, to impact disease risk (25–27). European ancestry GWAS have also previously hinted at a distinct association signal at this locus, mapping to the non-coding *CDKN2B-AS1* (*ANRIL*) transcript (4). Through exact conditioning, our transancestral meta-analysis has demonstrated that the association at this locus can be delineated by two distinct signals (Supplementary Material, Fig. S3), both of which map to the 12 kb intergenic recombination interval described above (rs10965246, MANTRA log_10_BF = 37.45, *P*= 8.4 × 10^−40^; rs10757282, MANTRA log_10_BF = 10.31, *P*= 2.0 × 10^−12^). Furthermore, our results highlight that these two index variants are sufficient to fully explain the association across the locus, including that previously localized to *CDKN2B-AS1* (maximum MANTRA log_10_BF = 1.88, *P*= 0.21, rs10811649).

### Evaluation of heterogeneity in association signals between ancestry groups

We next sought to evaluate the evidence for heterogeneity in allelic effects between studies for the index variants for the seven distinct association signals across the four loci on the basis of the transancestral meta-analysis (Materials and Methods). We observed no substantial differences in allelic OR, within or between ancestry groups, for any association signal (assessed by MANTRA log_10_BF of heterogeneity or Cochran's *Q* statistic from the fixed-effects meta-analysis). Any apparent differences in the magnitude of an association signal between ancestral clades, as measured by means of the log_10_BF or *P*-value, can be explained by differences in the allele frequency of the index variant between the diverse populations contributing to the meta-analysis (Supplementary Material, Table S3). For example, the index variant rs2237897, mapping to the *KCNQ1* locus, demonstrates a stronger signal of association after conditional analysis in the EAsia clade (MANTRA log_10_BF = 5.55, fixed-effects *P*= 2.0 × 10^−7^) than the Eur-MexAm-SAsia clade (MANTRA log_10_BF = 4.04, fixed-effects *P*= 4.4 × 10^−6^), despite much smaller total sample size. However, the minor allele is at much lower frequency in European, Mexican American and South Asian ancestry populations (MAF = 0.05) than in those of East Asian descent (MAF = 0.35), resulting in reduced power to detect association for the same allelic effect size.

### Localization of variants driving T2D association signals

We next constructed ‘credible sets’ of SNPs (27) that are most likely to drive each of the seven distinct signals at the four loci on the basis of their posterior probability of driving the association (*π*_C_) from the MANTRA transancestral meta-analysis (Materials and Methods, Table 2, Supplementary Material, Table S4). Assuming that the variant driving the association signal has been imputed from the 1000G reference panel, the probability that it will be contained in the 99% credible set is 0.99. Smaller credible sets, in terms of the number of SNPs they contain, or the genomic interval that they cover, thus correspond to fine-mapping at higher resolution. To assess the improvements in the resolution of fine-mapping offered by transancestral meta-analysis, we compared the properties of the 99% credible set for each of the seven distinct association signals obtained from: (i) studies in the EAsia clade only; (ii) studies in the Eur-MexAm-SAsia clade only and (iii) studies from all populations, combining the two clades with the single African American study. Note that we have not reported summary statistics for the 99% credible sets for the African American study alone because the small sample size makes comparison of fine-mapping intervals with the EAsia and Eur-MexAm-SAsia clades meaningless.

**Table 2.** Properties of the 99% credible sets of SNPs underlying each distinct association signal on the basis of meta-analyses of: (i) GWAS in the EAsia clade only; (ii) GWAS in the Eur-MexAm-SAsia clade only and (iii) GWAS from all ancestry groups

Locus	Index SNP	EAsia meta-analysis		Eur-MexAm-SAsia meta-analysis		Transancestral meta-analysis		
		SNPs	Distance (bp)	SNPs	Distance (bp)	SNPs	Distance (bp)	Interval (b37)
*IGF2BP2*	rs11705729	51	52 598	40	39 163	36	31 027	185 503 456–185 534 482
*CDKAL1*	rs9368222	15	32 429	8	40 463	5	12 330	20 675 792–20 688 121
*CDKN2A-B*	rs10965246	7	1556	7	2178	5	1371	22 132 698–22 134 068
	rs10757282	26	50 986	7	5861	7	4435	22 133 251–22 137 685
*KCNQ1*	rs231353	289	462 551	6	38 477	3	17 549	2 691 471–2 709 019
	rs233448	24	26 115	11	21 685	11	20 273	2 837 625–2 857 897
	rs2237897	9	18 886	53	474 488	3	197	2 858 440–2 858 636

**Figure 1.**
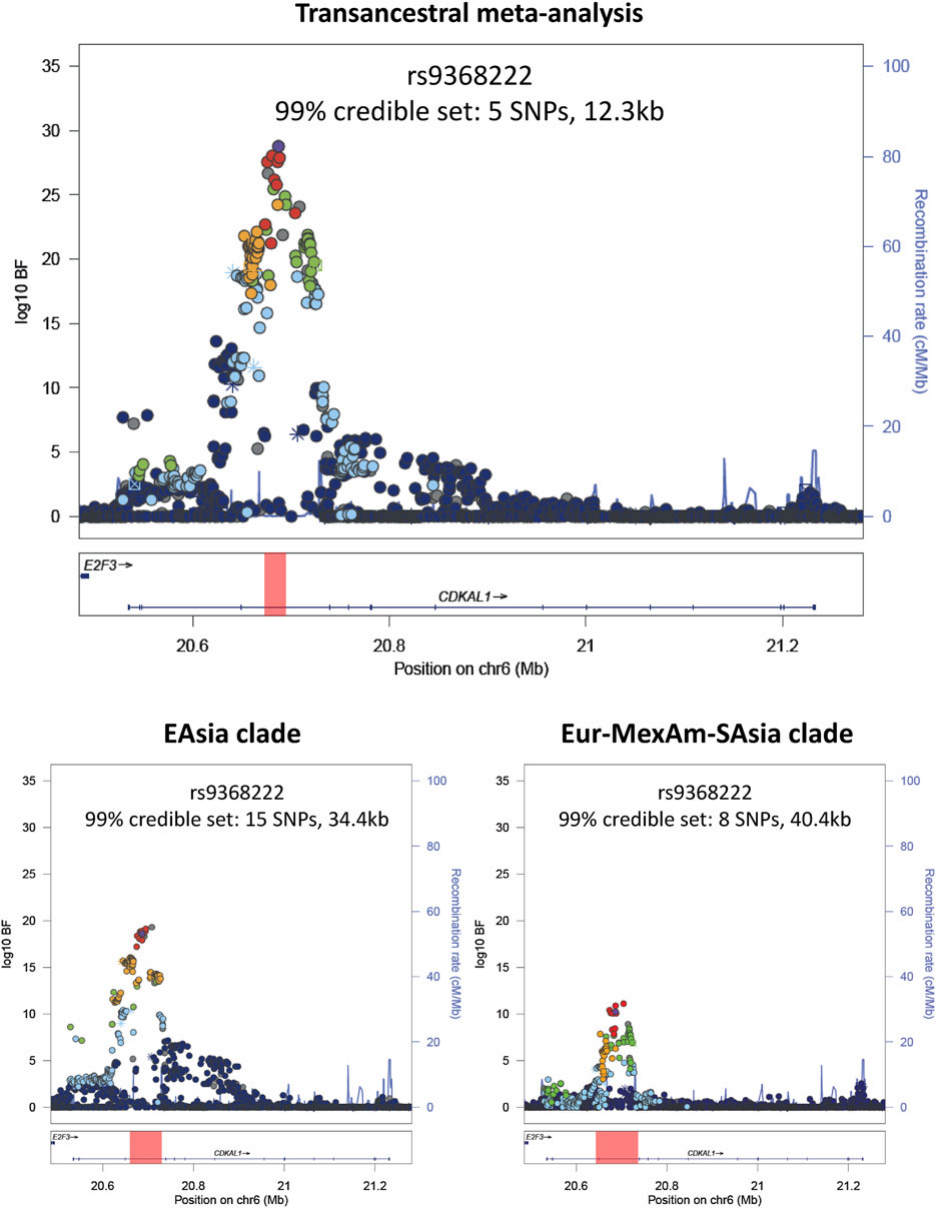
Fine-mapping of the association signal at the *CDKAL1* locus on the basis of transancestral meta-analysis of GWAS from all ancestry groups (top) and GWAS in the EAsia and Eur-MexAm-SAsia ancestral clades only (bottom). Each point represents a SNP passing quality control in the transancestral meta-analysis, plotted with their log_10_BF as a function of genomic position (NCBI Build 37). In each plot, the index SNP is represented by the purple symbol. The colour coding of all other SNPs indicates LD with the index SNP (estimated from 1000 Genomes Project reference haplotypes by EUR *r*^2^ for the transancestral meta-analysis and Eur-Mex-SAsia clade, and by ASN *r*^2^ for the EAsia clade): red *r*^2^ ≥ 0.8; gold 0.6 ≤ *r*^2^ < 0.8; green 0.4 ≤ *r*^2^ < 0.6; cyan 0.2 ≤ *r*^2^ < 0.4; blue *r*^2^ < 0.2; grey *r*^2^ unknown. The shape of the plotting symbol corresponds to the annotation of the SNP: upward triangle for framestop or splice; downward triangle for non-synonymous; square for synonymous or UTR; and circle for intronic or non-coding. Recombination rates are estimated from Phase II HapMap and gene annotations are taken from the UCSC genome browser. The genomic interval covered by the 99% credible set of variants for the association signal from the transancestral and ancestry-specific meta-analyses are highlighted by the red bar.

For each of the seven distinct association signals, fine-mapping resolution was improved after transancestral meta-analysis when compared with either ancestral clade, in terms of the number of SNPs reported in the credible set and/or the genomic interval that they cover (Table 2). These improvements in resolution could occur as a result of increased sample size, or because of differences in the structure of LD between diverse populations, but distinguishing between these possibilities is not straightforward. One approach is to quantify the extent of LD variation at a locus between pairs of populations by means of the varLD statistic (28). Using CEU, YRI and CHB + JPT reference haplotypes from the International HapMap Consortium (14) as representative of populations of European, African and East Asian ancestry, respectively, the *CDKAL1* locus has the greatest extent of LD variation among those investigated here, and thus would be expected to be most amenable to transancestral fine-mapping (28). At this locus, the 99% credible set for the association signal after transancestral meta-analysis included just five SNPs mapping to 12.3 kb, compared with 15 SNPs mapping to 34.4 kb in the EAsia clade, and eight SNPs mapping to 40.4 kb in the Eur-MexAm-SAsia clade (Fig. 1). The transancestral credible set corresponds to the overlap of SNPs from the two ancestral clades, and represents those that are in strong LD with the index variant (rs9368222) in East Asian and European descent populations. In contrast, the extent of variation in LD between CEU, YRI and CHB + JPT reference haplotypes from Phase II HapMap is lower at the *IGF2BP2* locus (28), where the improvement in the resolution of fine-mapping after transancestral meta-analysis is less apparent (Table 2). Variants in the 99% credible set for this association signal after transancestral meta-analysis are in strong LD with the lead SNP in both East Asian and European descent populations (CEU and CHB + JPT *r*^2^ > 0.7), so there is less gain for fine-mapping over the EAsia and Eur-MexAm-SAsia clades.

After transancestral meta-analysis, the most precise localization was observed for two of the association signals at the *KCNQ1* locus, indexed by rs2237897 (3 SNPs mapping to 197 bp of the narrow intronic recombination interval) and rs231353 (3 SNPs mapping to 17.5 kb of *KCNQ1-OT1*). The 99% credible sets for both association signals at the *CDKN2A-B* locus include a total of 12 non-overlapping SNPs mapping to the same <5 kb interval. We interrogated the 99% credible sets for all seven distinct association signals at the four loci for functional annotation. Despite the high-resolution of fine-mapping for all but the *IGF2BP2* association signal, the credible sets do not include any coding variants. These data are thus consistent with previous genome-wide reports that association signals for complex human traits at GWAS loci are most likely to be mediated through gene regulation (29,30).

### Regulatory mechanisms through which credible set variants influence T2D susceptibility

**Figure 2.**
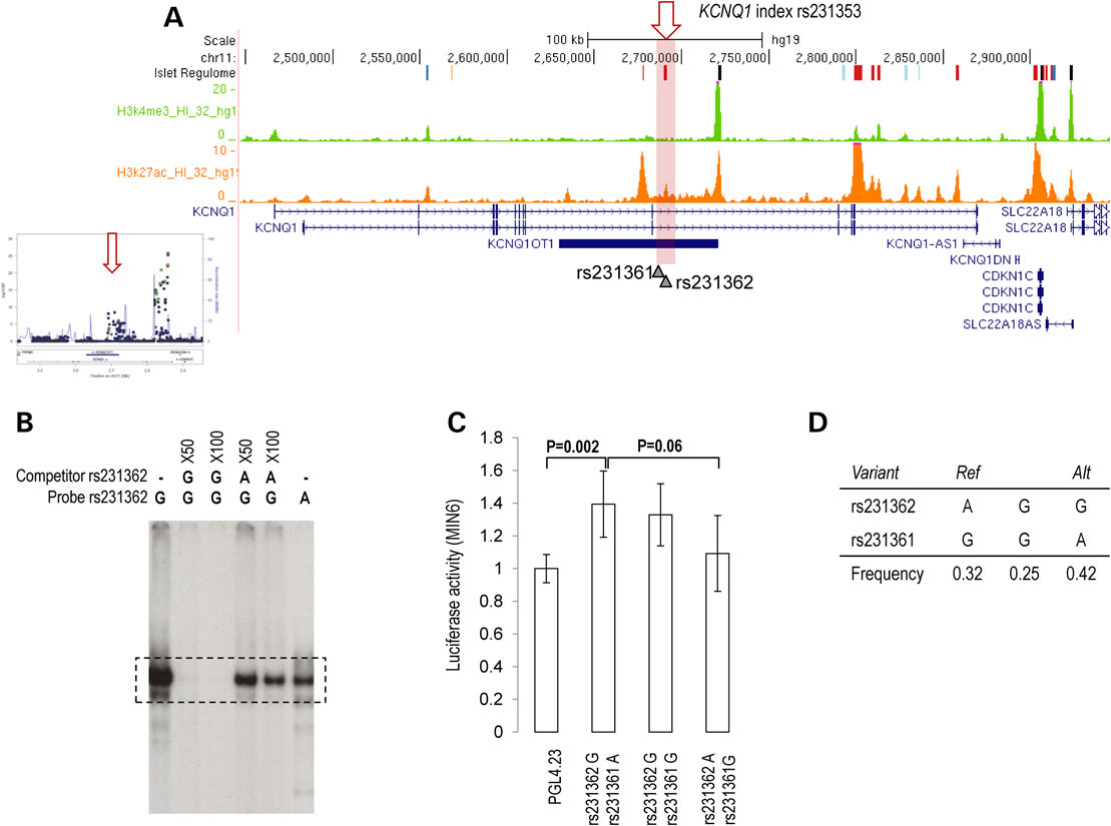
Allele-specific enhancer function at the *KCNQ1* locus. (**A**) At the *KCNQ1* association signal indexed by rs231353 (mapping to *KCNQ1-OT1*), 99% credible set variants rs231362 and rs231361 overlap a human pancreatic islet predicted enhancer characterized by an enrichment of the active histone modification H3K27ac. (**B**) Electrophoretic mobility shift assay, performed with MIN6 β-cells nuclear extracts, indicates allele-specific protein complex binding to the rs231362 variant. Allele G of the variant rs231362 allows the binding of a protein complex which does not disappear after pre-incubation with an excess of rs231362-A unlabelled oligonucleotide probe (competitor). (**C**) Luciferase assay shows reduced enhancer activity for haplotypes bearing the allele A compared with allele G of rs231362 in MIN6 β-cells. The data are presented as mean ± standard deviation. Three independent experiments were performed in triplicate, and *P*-values were calculated by a two-sided Student's *t*-test. (**D**) Allele frequencies for credible set variants rs231362 and rs231361.

Recent reports have demonstrated a relationship between T2D-associated variants, genome-wide, and transcriptional enhancer activity, particularly in human pancreatic islets, liver cells, adipose tissue and muscle (29–32). However, the precise biological processes by which these variants impact on disease susceptibility at most GWAS loci remain obscure. Given the primary physiological impact on T2D susceptibility of the four loci considered here via β-cell dysfunction (4), we explored potential mechanisms through which the effects of the seven distinct association signals are mediated by overlapping 99% credible set variants with regions of predicted regulatory function in human pancreatic islets (32) (Materials and Methods). We observed that credible set variants for four association signals (three at *KCNQ1* and one at *CDKAL1*) map to predicted tissue-specific enhancers in human pancreatic islets, suggesting that they may play a role in gene regulation (Fig. 2, Supplementary Material, Figs. S4–S6). These variants included: rs231362 and rs231361 (at the *KCNQ1* association signal indexed by rs231353, total *π*_C_ = 0.359); rs234866 (at the *KCNQ1* association signal indexed by rs233448, *π*_C_ = 0.048); rs2237897, rs2237896 and rs74046911 (the entire credible set at the *KCNQ1* association signal indexed by rs2237897, total *π*_C_ = 0.990); and rs9348441 (at the lone *CDKAL1* association signal indexed by rs9368222, *π*_C_ = 0.120).

To test a potential regulatory role of these variants, we first scanned the enhancer region for potential transcription factor binding sequences (Materials and Methods). We determined that rs231362, at the *KCNQ1* association signal indexed by rs231353, disrupts a bHLH-like motif. Within the large super-family of bHLH transcription factors, the best aligned score was found for the recognition site of BHLHE40. However, we cannot exclude the possibility of *in vivo* binding of other proteins from the same family at this site. Electrophoretic mobility shift assay (EMSA), performed using nuclear extracts obtained from the insulinoma mouse β cell line MIN6 (Materials and Methods), confirmed that rs231362 alters the binding of a protein complex *in vitro* (Fig. 2). We next created allele-specific luciferase reporter constructs of the predicted regulatory region overlapped by this association signal, and measured enhancer activity in MIN6 cells (Materials and Methods). This experiment confirmed the enhancer potential of the genomic site, and revealed higher activity of the haplotype of T2D-risk alleles, *in cis*, at rs231362 and rs231361 (Fig. 2). At the remaining association signals, allele-specific episomal reporter assays tested in mouse MIN6 cells failed to demonstrate enhancer activity at overlapping sites (Supplementary Material, Figs. S4–S6). Taken together, these observations highlight rs231362 as a potential functional variant, and point to the alteration of pancreatic islet genome regulation as a possible mechanism through which the association signal indexed by rs231353 at the *KCNQ1* locus is mediated.

## Discussion

We have undertaken comprehensive transancestral fine-mapping of four established T2D susceptibility loci to localize potential causal variants for association signals in 22 086 cases and 42 539 controls from diverse populations. Our study has extended previous transancestral T2D GWAS meta-analyses (10) through 1000G imputation and conditional analyses to improve fine-mapping resolution of distinct association signals in these loci. We have confirmed previous reports of multiple distinct association signals mapping to/near *KCNQ1* and *CDKN2A-B*, which may reflect multiple causal variants acting in isolation or through their joint effects, *in cis*, on the same haplotype. However, for the first time, we have demonstrated that these distinct association signals are shared across ancestry groups, with no evidence of heterogeneity in allelic effects on T2D risk between populations for index SNPs, despite substantial variability in allele frequencies.

The utility of transancestral fine-mapping relies on the assumption that causal variants are shared across diverse populations. The lack of heterogeneity in allelic effects on T2D susceptibility between populations for distinct association signals at the four loci considered in this study is consistent with this assumption. Previous evidence of the transferability of T2D association signals across diverse populations (6–10) suggests that many established common variant loci for the disease will also be amenable to transancestral fine-mapping. Future discovery efforts, with imputation up to larger, higher-density reference panels with improved coverage across the MAF spectrum, would be expected to identify lower frequency association signals that are more likely to be ancestry- or population-specific, and thus unlikely to benefit from fine-mapping across diverse populations.

The resolution of fine-mapping (assessed by credible set size) will depend, crucially, on the extent of differences in the structure of LD between populations contributing to the transancestral meta-analysis (12). We observed the most precise localization of causal variants for the T2D association signal mapping near *CDKAL1*, which has the greatest difference in LD structure between populations of European, East Asian and African ancestry among those loci considered in our study (28). However, even at the *IGF2BP2* locus, where differences in LD between populations are less well defined, increased sample size in the transancestral meta-analysis offered improved resolution over ancestry-specific fine-mapping by magnifying even small deviations in the correlation of SNPs with the causal variant. We would expect, therefore, that transancestral fine-mapping would enable improved localization of T2D association signals across common variant GWAS loci, with further enhancements obtained through inclusion of additional African (American) descent populations, where the extent of LD is minimized.

Our fine-mapping experiment provided no evidence that association signals at the four susceptibility loci are driven by coding variants. Our data are thus consistent with previous genome-wide reports that association signals for T2D susceptibility are most likely to act via gene regulation (29,30). Here, using *KCNQ1* as an exemplar, we have demonstrated how genetic fine-mapping and genomic annotation can be used to highlight potential causal regulatory elements in disease-relevant tissues, thereby providing insight into the mechanisms through which association signals are mediated, and routes to understand the underlying biology of specific loci through directed functional experimentation. At this locus, our results highlight rs231362 as having a gain-of-function effect on a pancreatic islet enhancer element residing in intron 11 of the *KCNQ1* gene and overlapping the *KCNQ1-OT1* non-coding transcript, a region previously demonstrated to harbour tissue-specific active enhancers in mouse (33). While more experiments are needed to characterize the protein complex binding this regulatory element, we determined that rs231362 alters a bHLH-like motif. Several bHLH transcription factors are expressed in human pancreatic islets, including key islet regulators such as NEUROD1 (34). The best alignment score was found for the recognition site of the bHLH transcription factor BHLHE40, a protein expressed in human pancreatic islets and shown to play a role during the specification of pancreatic endocrine progenitor cells (35). However, the biological role of BHLHE40 in adult pancreatic islets remains unclear.

*KCNQ1* encodes for the voltage-gated K+ channel Kv7.1 in pancreatic β-cells. Over-expression of *KCNQ1* in cultured MIN6 cells has been shown to decrease glucose induced insulin secretion (36), and is thus in keeping with a gain-of-function regulatory mechanism in T2D susceptibility. Moreover, inhibition of Kv7.1 in β-cells has been previously demonstrated to increase exocytosis and secretion of insulin (37), and patients with loss-of-function mutations in *KCNQ1* exhibit increased insulin secretion (38). Although further functional experimentation, beyond the scope of this study, will be required to definitively establish the gene target of the regulatory element overlapping variants driving the *KCNQ1-OT1* association signal, these data point to *KCNQ1* as a possible candidate.

At the remaining association signals, episomal reporter assays performed in mouse MIN6 cells failed to demonstrate enhancer activity at sites overlapping credible set variants. While episomal assays cannot recapitulate the natural genomic and chromatin context, approaches such as genome-editing (39) could unmask a possible effect of these variants in their *cis*-regulatory milieu and enable isolation of their impact on β-cell gene expression and function.

In conclusion, we have demonstrated that transancestral meta-analysis of GWAS from diverse populations can be used to localize variants most likely to drive distinct association signals at T2D susceptibility loci. By integrating genetic fine-mapping with genomic information from diabetes-relevant tissues, we have demonstrated the utility of this approach for elucidating the mechanisms through which the effects of T2D association signals at GWAS loci on disease susceptibility are mediated. Our study and analytical protocols provide a prototype for future transancestral fine-mapping of T2D susceptibility loci, genome-wide. These efforts will be further enhanced by the release of larger, ancestry-specific imputation reference panels that incorporate reference haplotypes from a wider spectrum of global populations, and improved functional and regulatory genomic annotation, thus promising an exciting opportunity to explicate the, as yet, poorly understood pathophysiology of the disease.

## Materials and Methods

### Ethics statement

All human research was approved by the relevant institutional review boards, and conducted according to the Declaration of Helsinki. All participants provided written informed consent.

### Study-level analysis

Sample and SNP quality control was undertaken in each study (Supplementary Material, Table S1). In each of the four loci, the clean GWAS scaffold was then imputed up to the 1000 Genomes Project (Phase 1 integrated, all ancestries, March 2012 release) reference panel (15). Well-imputed variants, defined by IMPUTEv2 (16) info >0.4 or minimac (17) *r*^2^ > 0.3, were tested for association with T2D in a logistic regression framework under an additive model after adjustment for study-specific covariates (Supplementary Material, Table S2), including principal components to adjust for population structure. Under the assumption that the underlying causal variants for association signals at these loci are common and shared across ancestry groups, SNPs with MAF <1% were excluded from downstream analyses.

### Transancestral meta-analysis

Association summary statistics for each SNP were combined across studies using two complementary approaches: (i) a fixed-effects meta-analysis implemented in GWAMA (40) and (ii) a Bayesian hybrid of fixed- and random-effects meta-analysis, as implemented in MANTRA (19). Meta-analyses were performed first across studies within each of the EAsia and Eur-MexAm-SAsia ancestral clades (Supplementary Material, Fig. S1). Subsequently, meta-analyses were performed across all populations, bringing together the AfAm study with those from the EAsia and Eur-MexAm-SAsia ancestral clades. SNPs passing quality control in <80% of the total sample size (*N*≥ 51 700) were excluded from the transancestral meta-analysis.

The fixed-effects meta-analysis was performed by combining allelic effect sizes across studies under an inverse-variance weighting scheme (40). Genome-wide significance was defined by the standard threshold of *P*< 5 × 10^−8^. Heterogeneity in allelic effects was assessed by means of Cochran's *Q* statistic (41).

MANTRA was developed specifically for the purposes of transancestral fine-mapping, and allows for heterogeneity in allelic effects between ancestry groups arising as a result of differences in the structure of LD with the causal variant between diverse populations. MANTRA assigns studies to clusters according to a Bayesian partition model of relatedness between them, defined by pair-wise genome-wide mean allele frequency differences (Supplementary Material, Fig. S1). Genome-wide significance was defined by a threshold of log_10_BF ≥ 6, which has been demonstrated, by simulation, to be approximately equivalent to *P*< 5 × 10^−8^ under a fixed-effects model (19,20). MANTRA also provides an assessment of the evidence of heterogeneity in allelic effects by means of a BF, calculated by comparing a model where all studies are assigned to the same cluster, with one where the number of clusters is unconstrained.

### Identification of distinct association signals

We identified ‘index SNPs’ to represent distinct signals of association attaining genome-wide significance (MANTRA log_10_BF ≥ 6 and *P*< 5 × 10^−8^) at each locus through a series of conditional analyses, described below. Conditional analyses were performed in each study, testing for T2D association with well-imputed variants in a logistic regression framework under an additive model after adjustment for study-specific covariates (Supplementary Material, Table S2), and inclusion of genotypes at other index variants at the locus as additional covariates. Association summary statistics for each SNP were then combined across studies by means of a fixed-effects meta-analysis and MANTRA.

At the *IGF2BP2* locus, we included genotypes at the lead SNP (rs11705729) from the transancestral meta-analysis as an additional covariate in the logistic regression model, and no variants attained at the locus genome-wide significance after conditioning. The strongest residual association signal in conditional analysis was achieved by rs1540390 (log_10_BF = 0.98, *P*= 0.012). We concluded that there is one common variant association signal mapping to the *IGF2BP2* locus, indexed by rs11705729. Subsequent fine-mapping analyses were undertaken on the basis of the unconditional transancestral meta-analysis at this locus.

At the *CDKAL1* locus, we included genotypes at the lead SNP (rs9368222) from the transancestral meta-analysis as an additional covariate in the logistic regression model, and no variants at the locus attained genome-wide significance after conditioning. The strongest residual association signal was achieved by rs2328574 (log_10_BF = 1.76, *P*= 0.027). We concluded that there is one common variant association signal mapping to the *CDKAL1* locus, indexed by rs936822. Subsequent fine-mapping analyses were undertaken on the basis of the unconditional transancestral meta-analysis at this locus.

At the *CDKN2A-B* locus, we first included genotypes at the lead SNP (rs10965248) from the transancestral meta-analysis as an additional covariate in the logistic regression model, and multiple variants at the locus attained genome-wide significance (Supplementary Material, Fig. S3). The strongest residual association signal was attained by rs10757282 (log_10_BF = 10.31, *P*= 2.0 × 10^−12^). We next included genotypes at both rs10965248 and rs10757282 as additional covariates in the logistic regression model, and no variants at the locus attained genome-wide significance after this second round of conditioning. The strongest residual association signal was attained by rs10811649 (log_10_BF = 1.88, *P*= 0.21). We concluded that there are two distinct signals of association mapping to the *CDKN2A-B* locus. Subsequent fine-mapping analyses for distinct common variant association signals at this locus were thus based on: (i) conditional analysis after adjustment for genotypes at rs10757282 as an additional covariate (index variant rs10965246) and (ii) conditional analysis after adjustment for genotypes at rs10965248 as an additional covariate (index variant rs10757282).

At the *KCNQ1* locus, visual inspection of the signal plot for the transancestral meta-analysis revealed three SNPs that were not in LD with each other in any ancestry group (*r*^2^ < 0.04), but all attaining genome-wide significance: rs2237896, rs231353 and rs234864 (Supplementary Material, Fig. S2). We thus included genotypes at all three of these SNPs as additional covariates in the logistic regression model, and no variants at the locus attained genome-wide significance after conditioning. The strongest residual association signal was achieved by rs233448 (log_10_BF = 3.38, *P*= 2.1 × 10^−5^). We concluded that there are three distinct common variant association signals mapping to the *KCNQ1* locus. Subsequent fine-mapping analyses for distinct association signals at this locus were thus based on: (i) conditional analysis after adjustment for genotypes at rs2237896 and rs231353 as additional covariates (index variant rs233448); (ii) conditional analysis after adjustment for genotypes at rs2237896 and rs234864 (index variant rs231353) and (iii) conditional analysis after adjustment for genotypes at rs231353 and rs234864 (index variant rs2237897).

### Credible set construction

We calculated the posterior probability that the *j*th variant, *π*_C*j*_, is driving a distinct association signal by

$$\pi_{Cj}=\frac{\Lambda_{j}}{\sum_{k}\Lambda_{k}},$$

where the summation is over all variants in the locus. In this expression, Λ*_j_* is the MANTRA BF in favour of association from the transancestral meta-analysis. In loci with multiple distinct signals of association (*KCNQ1* and *CDKN2A-B*), results are presented from conditional meta-analysis as described above. In loci with a single association signal (*IGF2BP2* and *CDKAL1*), results are presented from unconditional meta-analysis. A 99% credible set (27) was then constructed by: (i) ranking all variants according to their BF, Λ*_j_* and (ii) including ranked variants until their cumulative posterior probability exceeds 0.99.

For each association signal, credible sets were constructed on the basis of the MANTRA BF in favour of association on the basis of the following meta-analyses: (i) studies within the EAsia ancestral clade only; (ii) studies within the Eur-MexAm-SAsia ancestral clade only and (iii) all studies across ancestry groups.

### Genomic annotation and functional study of credible set variants

We overlapped annotations obtained from human pancreatic islets (32) with variants in 99% credible sets using bedtools v2.17.0 (42). Scanning for motifs and motif annotation was performed using HOMERv4.4 (43) with default settings. The selected human islet predicted regulatory regions of length 1.5–1.9 kb were PCR-amplified from human genomic DNA with Phusion High-Fidelity DNA Polymerase (New England Biolabs), cloned into pENTR/D-TOPO (Invitrogen, catalogue number K2400-20) and shuttled into Gateway-adapted PGL4.23 (44) with Gateway LR Clonase Enzyme Mix (Invitrogen, catalogue number 11791-100). The plasmids were modified by site-directed mutagenesis (QuickChange; Stratagene, Santa Clara, CA, USA) to produce the common and rare genotype of the associated variants and to reproduce risk and protective haplotypes. Correct mutagenesis was confirmed by Sanger sequencing.

Mouse β-cells (MIN6) were co-transfected in triplicate wells with pGL4.23-regulatory region and pRL using Lipofectamine 2000 (Invitrogen), and luciferase activity was measured after 48 h. Results were expressed as luciferase:renilla ratios in vectors carrying putative regulatory regions, relative to the ratio in empty PGL4.23 vector. Statistical significance was assessed using a two-sided Student's *t*-test across all experiments.

EMSA was performed with mouse MIN6 β-cell nuclear extracts as previously described (45). The sequences of oligonucleotides used in this assay to test both genotypes of the credible set variant rs231362 were:

rs231362: A 5′-GATCTTTGACCCTGCACATGACGGGCGAG-3́; and
rs231362: G 5′-GATCTTTGACCCTGCACGTGACGGGCGAG-3́.

## Supplementary Material

Supplementary Material is available at *HMG* online.

## Funding

This work was supported by Action on Hearing Loss (G51); American Federation for Aging; Einstein Glenn Center; Association Francaise des Diabetiques; British Heart Foundation (SP/04/002); CARDIOMED BSC0122(6)-CSIR, India; Centre Hospitalier Universitaire Poitiers; CNAMTS; Endocrinology-Diabetology Department of the Corbeil-Essonnes Hospital; European Union (FP7 EpiMigrant, 279143); Focused Investment Scheme of the Chinese University of Hong Kong; Fondation de France; Hong Kong Foundation for Research and Development in Diabetes; Genome Canada; Génome Quebec; Hong Kong Government Research Grant Committee Central Allocation Scheme (CUHK 1/04C), Innovation and Technology Fund (ITS/487/09FP and ITS/130/11), and the Research Grants Council Theme-based Research Scheme (T12-402/13N); Japan Society for the Promotion of Science (KAKENHI 23710228); Medical Research Council (G0601966, G0700931); Ministerio de Economía y Competitividad (BFU2014-58150-R); Ministry of Education, Culture, Sports, Science and Technology of Japan; National Center for Global Health and Medicine (NCGM); National Institute of Aging (PO1AG027734, R01AG046949, 1R01AG042188, P30AG038072, R014AG028872); National Institutes of Health (K24-DK080140, U01-DK085526, R01-MH101820, P60-DK20595, U01-DK085501, R01-HL102830, U01-HG005773, R01-MH090937, R01-HL102830, R01-HG000376, R01-DK062370, R01-DK073541, U01-DK085524, U01-DK085545, U01-DK085584, U01- DK105535); National Institute for Health Research (RP-PG-0407-10371); National Institute for Health Research (NIHR) Comprehensive Biomedical Research Centre, Imperial College Healthcare NHS Trust; National Medical Research Program, Singapore; National Research Foundation of Korea (NRF-2012R1A2A1A03006155, 2012R1A3A2026438, 2013M3A9C4078158, 2015R1A5A6001906); Program for Promotion of Fundamental Studies in Health Sciences, National Institute of Biomedical Innovation Organization (NIBIO); Wellcome Trust (WT084723, WT090532, WT098017, WT098051). The Jackson Heart Study is supported by contracts HHSN268201300046C, HHSN268201300047C, HHSN268201300048C, HHSN268201300049C, HHSN268201300050C from the National Heart, Lung, and Blood Institute and the National Institute on Minority Health and Health Disparities. Funding to pay the Open Access publication charges for this article was provided by the Wellcome Trust.

## Supplementary Material

Supplementary Data
