# Supplementary material for: Transancestral fine-mapping of four type 2 diabetes susceptibility loci highlights potential causal regulatory mechanisms
Source: Hum Mol Genet. 2016 Feb 23;25(10):2070–81. doi: 10.1093/hmg/ddw048 (PMC5062576; doi:10.1093/hmg/ddw048)
Supplement: Supplementary Data [file supp_25_10_2070__index.html]

Transancestral fine-mapping of four type 2 diabetes susceptibility loci highlights potential causal regulatory mechanisms — Transancestral fine-mapping of four type 2 diabetes susceptibility loci highlights potential causal regulatory mechanisms — Supplementary Data 

# Transancestral fine-mapping of four type 2 diabetes susceptibility loci highlights potential causal regulatory mechanisms

## Supplementary Data

Supplementary Data

- Supplementary Data - Pdf file
