# Supplementary material for: Transancestral fine-mapping of four type 2 diabetes susceptibility loci highlights potential causal regulatory mechanisms
Source: Hum Mol Genet. 2016 Feb 23;25(10):2070–81. doi: 10.1093/hmg/ddw048 (PMC5062576; doi:10.1093/hmg/ddw048)
Supplement: Supplementary Data [file supp_ddw048_ddw048supp.pdf]

Transancestral fine-mapping of four type 2 diabetes susceptibility loci highlights potential causal regulatory mechanisms

SUPPLEMENTARY MATERIAL

Momoko Horikoshi[1,2], Lorenzo Pasquali[3,4,5], Steven Wiltshire[1,2,†], Jeroen R Huyghe[6], Anubha Mahajan[1], Jennifer L Asimit[7], Teresa Ferreira[1], Adam E Locke[6], Neil R Robertson[1,2], Xu Wang[8], Xueling Sim[6,8], Hayato Fujita[9], Kazuo Hara[10], Robin Young[11], Weihua Zhang[12,13], Sungkyoung Choi[14], Han Chen[15,16], Ismeet Kaur[17], Fumihiko Takeuchi[18], Pierre Fontanillas[19], Dorothée Thuillier[20], Loic Yengo[20], Jennifer E Below[21], Claudia HT Tam[22], Ying Wu[23], T2D-GENES Consortium, Gonçalo Abecasis[6], David Altshuler[19,24,25,26,27,28], Graeme I Bell[29], John Blangero[30], Noél P Burtt[19], Ravindranath Duggirala[30], Jose C Florez[19,27,28,31], Craig L Hanis[21], Mark Seielstad[32,33], Gil Atzmon[34,35], Juliana CN Chan[22,36,37], Ronald CW Ma[22,36,37], Philippe Froguel[38,39], James G Wilson[40], Dwaipayan Bharadwaj[17,41], Josee Dupuis[16,42], James B Meigs[28,43], Yoon Shin Cho[44], Taesung Park[14,45], Jaspal S Kooner[12,46,47], John C Chambers[12,13,47], Danish Saleheen[48], Takashi Kadowaki[10,49], E Shyong Tai[8,50,51], Karen L Mohlke[23], Nancy J Cox[52], Jorge Ferrer[5,53,54], Eleftheria Zeggini[7], Norihiro Kato[18], Yik Ying Teo[8,55,56], Michael Boehnke[6,‡], Mark I McCarthy[1,2,57,‡], and Andrew P Morris[1,58,‡]

[1]Wellcome Trust Centre for Human Genetics, Nuffield Department of Medicine, University of Oxford, Oxford, UK. [2]Oxford Centre for Diabetes, Endocrinology and Metabolism, Radcliffe Department of Medicine, University of Oxford, Oxford, UK. [3]Department of Endocrinology, Germans Trias i Pujol University Hospital and Research Institute, Badalona, Spain. [4]Josep Carreras Leukaemia Research Institute, Badalona, Spain. [5]CIBER de Diabetes y Enfermedades Metabólicas Asociadas (CIBERDEM), Barcelona, Spain. [6]Department of Biostatistics and Center for Statistical Genetics, University of Michigan, Ann Arbor, Michigan, USA. [7]Department of Human Genetics, Wellcome Trust Sanger Institute, Hinxton, Cambridgeshire, UK. [8]Saw Swee Hock School of Public Health, National University of Singapore, National University Health System, Singapore, Singapore. [9]Department of Diabetes and Endocrinology, JR Tokyo General Hospital, Tokyo, Japan. [10]Department of Diabetes and Metabolic Diseases, Graduate School of Medicine, University of Tokyo, Tokyo, Japan. [11]Department of Public Health and Primary Care, Institute of Public Health, University of Cambridge, Cambridge, UK. [12]Department of Cardiology, Ealing Hospital NHS Trust, Southall, Middlesex, UK. [13]Department of Epidemiology and Biostatistics, Imperial College London, London, UK. [14]Interdisciplinary Program in Bioinformatics, Seoul National University, Seoul, Republic of Korea. [15]Department of Biostatistics, Harvard School of Public Health, Boston, Massachusetts, USA. [16]Department of Biostatistics, Boston University School of Public Health, Boston, Massachusetts, USA. [17]Genomics and Molecular Medicine, CSIR-Institute of Genomics & Integrative Biology, New Delhi, India. [18]Department of Gene Diagnostics and Therapeutics, Research Institute, National Center for Global Health and Medicine, Tokyo, Japan. [19]Program in Medical and Population Genetics, Broad Institute, Cambridge, Massachusetts, USA. [20]Integrative Genomics and Modelization of Metabolic Diseases CNRS UMR8199, Lille Institute of Biology, E.G.I.D - FR3508 European Genomics

Institute of Diabetes, Lille, France. [21]Human Genetics Center, School of Public Health, University of Texas Health Science Center at Houston, Houston, Texas, USA. [22]Department of Medicine and Therapeutics, Chinese University of Hong Kong, Hong Kong, China. [23]Department of Genetics, University of North Carolina, Chapel Hill, North Carolina, USA. [24]Department of Biology, Massachusetts Institute of Technology, Cambridge, Massachusetts, USA. [25]Department of Genetics, Harvard Medical School, Boston, Massachusetts, USA. [26]Department of Molecular Biology, Massachusetts General Hospital, Boston, Massachusetts, USA. [27]Diabetes Research Center (Diabetes Unit), Department of Medicine, Massachusetts General Hospital, Boston, Massachusetts, USA. [28]Department of Medicine, Harvard Medical School, Boston, Massachusetts, USA. [29]Departments of Medicine and Human Genetics, University of Chicago, Chicago, Illinois, USA. [30]Department of Genetics, Texas Biomedical Research Institute, Houston, Texas, USA. [31]Center for Human Genetic Research, Department of Medicine, Massachusetts General Hospital, Boston, Massachusetts, USA. [32]Blood Systems Research Institute, San Francisco, California, USA. [33]Department of Laboratory Medicine & Institute for Human Genetics, University of California, San Francisco, San Francisco, California, USA. [34]Department of Natural Science, University of Haifa, Haifa, Israel. [35]Departments of Medicine and Genetics, Albert Einstein College of Medicine, New York, USA. [36]Hong Kong Institute of Diabetes and Obesity, Chinese University of Hong Kong, Hong Kong, China. [37]Li Ka Shing Institute of Health Sciences, Chinese University of Hong Kong, Hong Kong, China. [38]Department of Genomics of Common Disease, School of Public Health, Imperial College London, London, UK. [39]Genomics and Molecular Physiology, CNRS (Institut de Biologie de Lille), Lille, France. [40]Department of Physiology and Biophysics, University of Mississippi Medical Center, Jackson, Mississippi, USA. [41]School of Biotechnology, Jawaharlal Nehru University, New Delhi, India. [42]National Heart, Lung, and Blood Institute's Framingham Heart Study, Framingham, Massachusetts, USA. [43]General Medicine Division, Massachusetts General Hospital, Boston, Massachusetts, USA. [44]Department of Biomedical Science, Hallym University, Chuncheon, Republic of Korea. [45]Department of Statistics, Seoul National University, Seoul, Republic of Korea. [46]National Heart and Lung Institute, Cardiovascular Sciences, Hammersmith Campus, Imperial College London, London, UK. [47]Imperial College Healthcare NHS Trust, Imperial College London, London, UK. [48]Department of Biostatistics and Epidemiology, Center for Non-Communicable Diseases, University of Pennsylvania, Philadephia, Pennsylvania, USA. [49]Department of Integrated Molecular Science on Metabolic Diseases, 22nd Century Medical and Research Center, The University of Tokyo, Tokyo, Japan. [50]Cardiovascular & Metabolic Disorders Program, Duke-NUS Graduate Medical School Singapore, Singapore. [51]Department of Medicine, Yong Loo Lin School of Medicine, National University of Singapore, National University Health System, Singapore. [52]School of Medicine, Vanderbilt University, Nashville, Tennessee, USA. [53]Genomic Programming of Beta-cells Laboratory, Institut d'Investigacions August Pi i Sunyer (IDIBAPS), Barcelona, Spain. [54]Department of Medicine, Imperial College London, London, UK. [55]Life Sciences Institute, National University of Singapore, Singapore. [56]Department of Statistics and Applied Probability, National University of Singapore, Singapore. [57]Oxford NIHR Biomedical Research Centre, Oxford University Hospitals Trust, Oxford, UK. [58]Department of Biostatistics, University of Liverpool, Liverpool, UK.

[‡]These authors jointly supervised this work.
[†]Deceased.

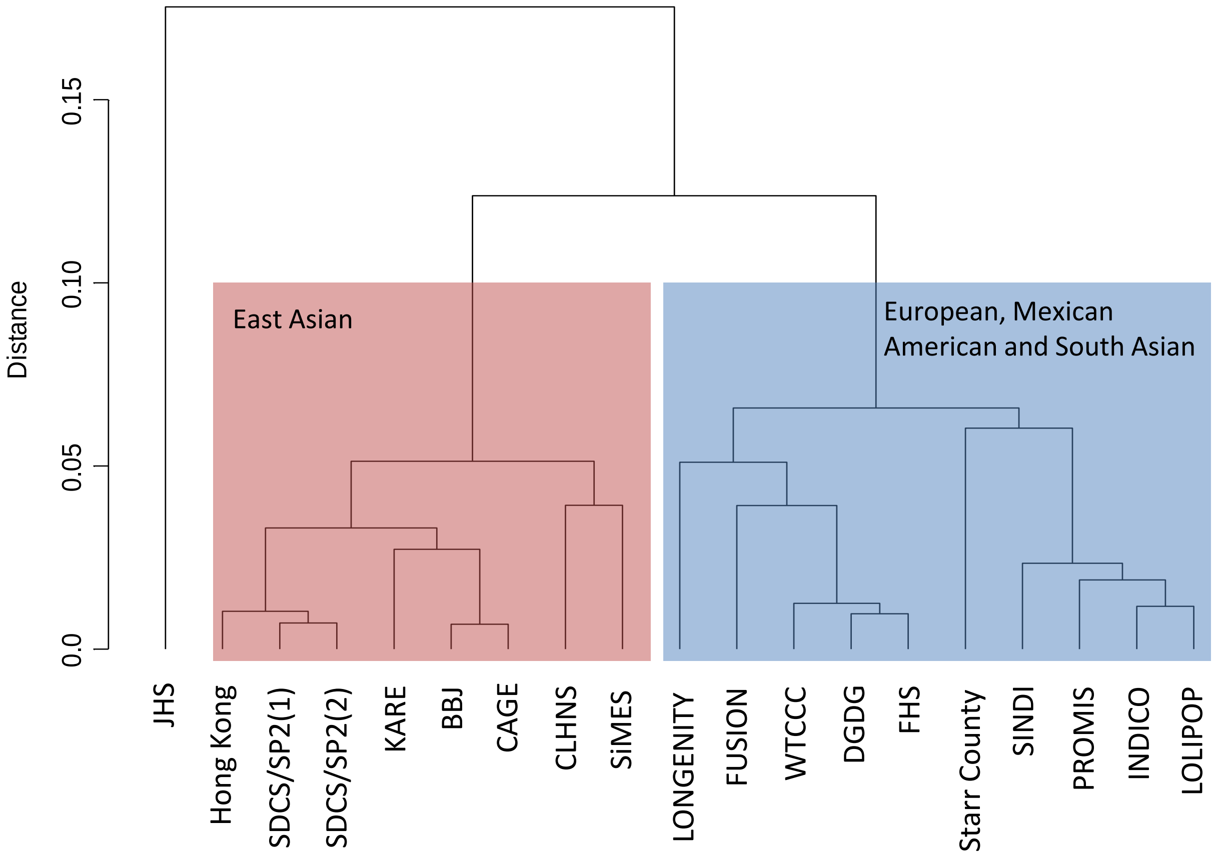

Figure S1. Dendogram to summarise relatedness between studies. The dendogram was constructed on the basis of pair-wise allele frequency differences between studies across the five loci. With the exception of JHS (African American), the studies fall into two "ancestral clades": East Asian (highlighted in pink); and European, Mexican American and South Asian (highlighted in blue).

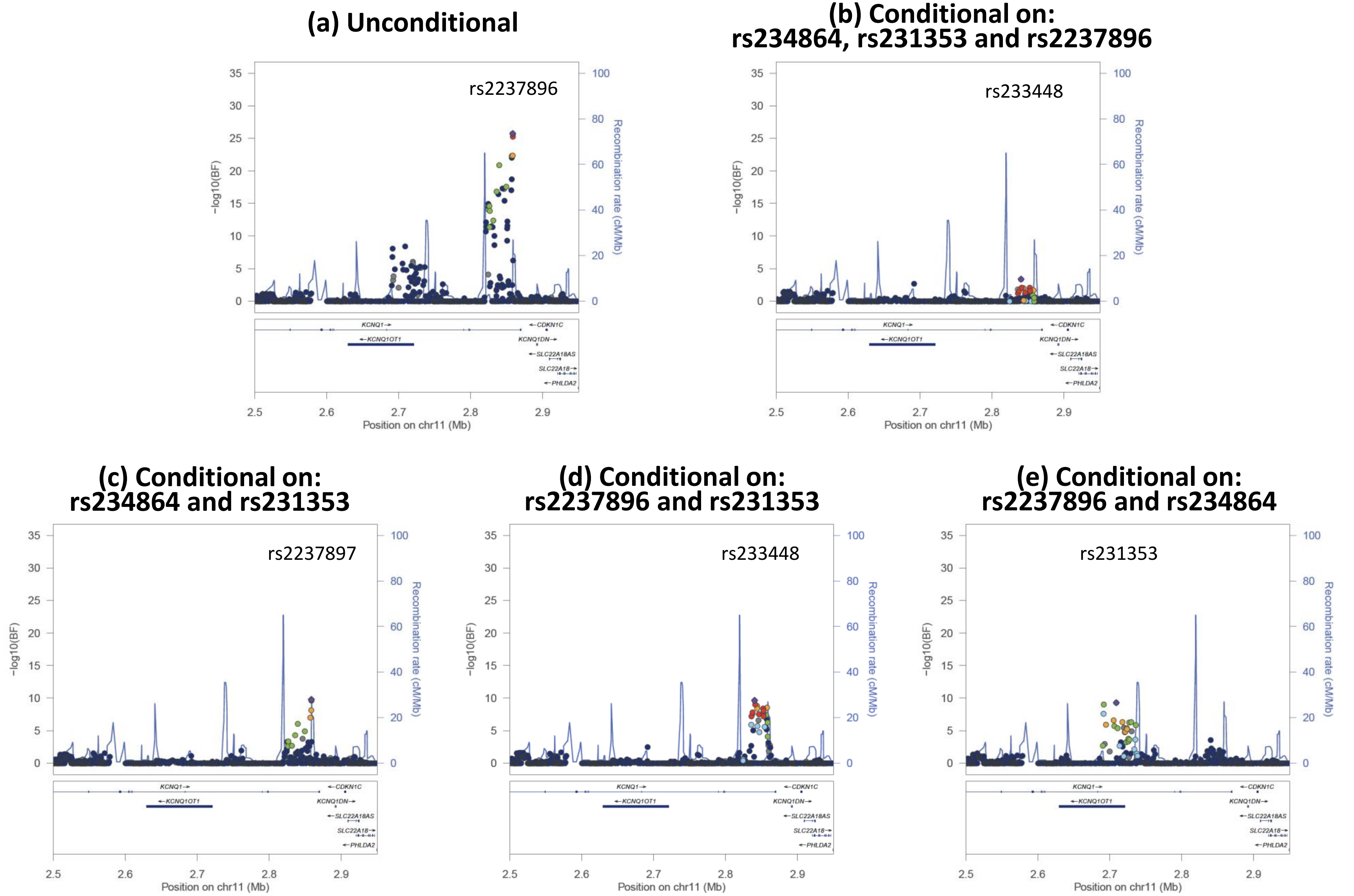

Figure S2. Signal plots at the *KCNQ1* locus, constructed on the basis of transancestral meta-analysis: (a) unconditional; (b) conditional on rs234864, rs231353 and rs2237896; (c) conditional on rs234864 and rs231353; (c) conditional on rs2237896 and rs231353; and (d) conditional on rs2237896 and rs234864. Each point represents a SNP passing quality control in the transancestral meta-analysis, plotted with their $\log_{10}$BF as a function of genomic position (NCBI Build 37). In each plot, the index SNP is represented by the purple symbol. The colour coding of all other SNPs indicates LD with the index SNP (estimated by EUR r^2 from 1000 Genomes Project reference haplotypes): red $r^2 \geq 0.8$; gold $0.6 \leq r^2 < 0.8$; green $0.4 \leq r^2 < 0.6$; cyan $0.2 \leq r^2 < 0.4$; blue $r^2 < 0.2$; grey r^2 unknown. Recombination rates are estimated from Phase II HapMap and gene annotations are taken from the UCSC genome browser.

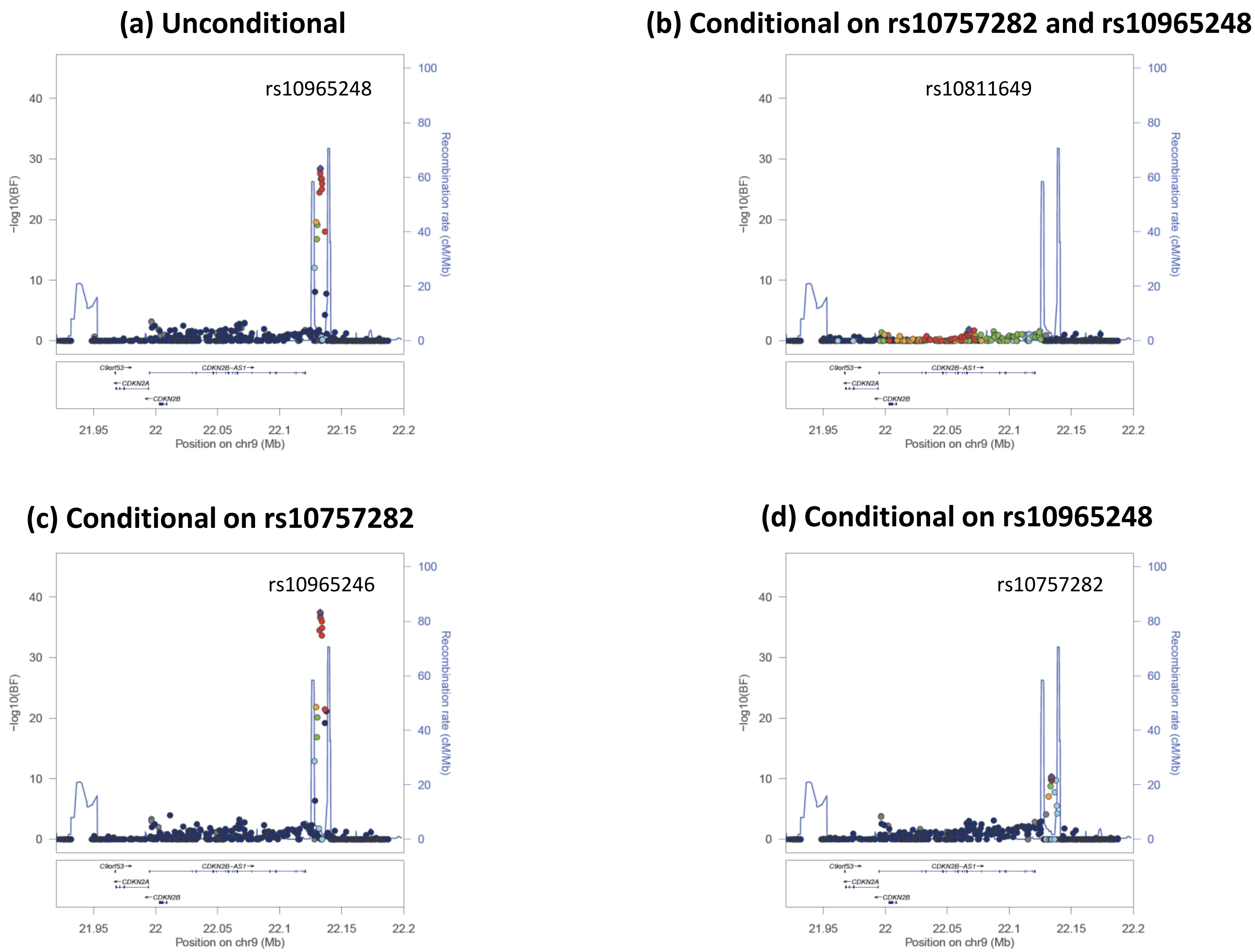

Figure S3. Signal plots at the *CDKN2A-B* locus, constructed on the basis of transancestral meta-analysis: (a) unconditional; (b) conditional on rs10757282 and rs10965248; (c) conditional on rs10757282; and (d) conditional on rs10965248. Each point represents a SNP passing quality control in the transancestral meta-analysis, plotted with their $\log_{10}$BF as a function of genomic position (NCBI Build 37). In each plot, the index SNP is represented by the purple symbol. The colour coding of all other SNPs indicates LD with the index SNP (estimated by EUR r^2 from 1000 Genomes Project reference haplotypes): red $r^2 \geq 0.8$; gold $0.6 \leq r^2 < 0.8$; green $0.4 \leq r^2 < 0.6$; cyan $0.2 \leq r^2 < 0.4$; blue $r^2 < 0.2$; grey r^2 unknown. Recombination rates are estimated from Phase II HapMap and gene annotations are taken from the UCSC genome browser.

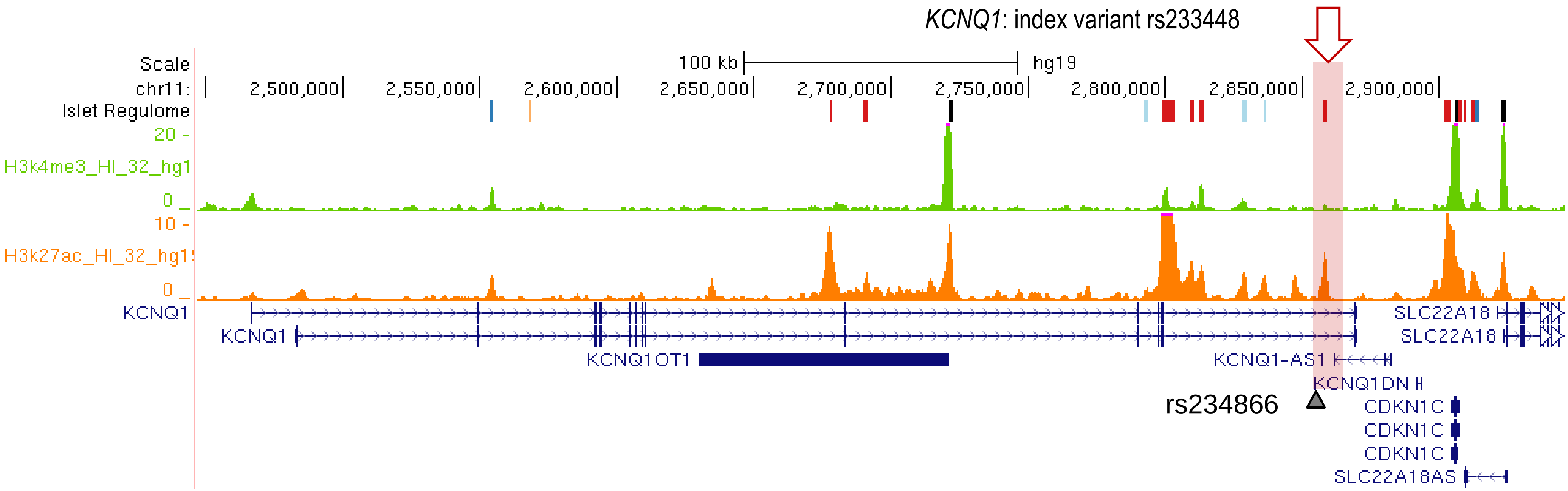

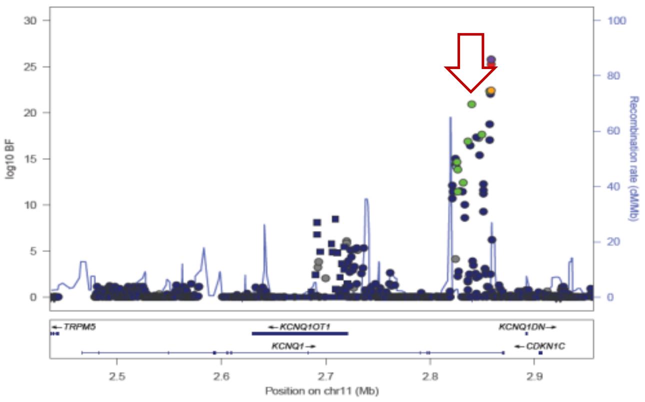

Variant	*Ref*	*Alt*
rs234866	G	A
Frequency	0.77	0.23

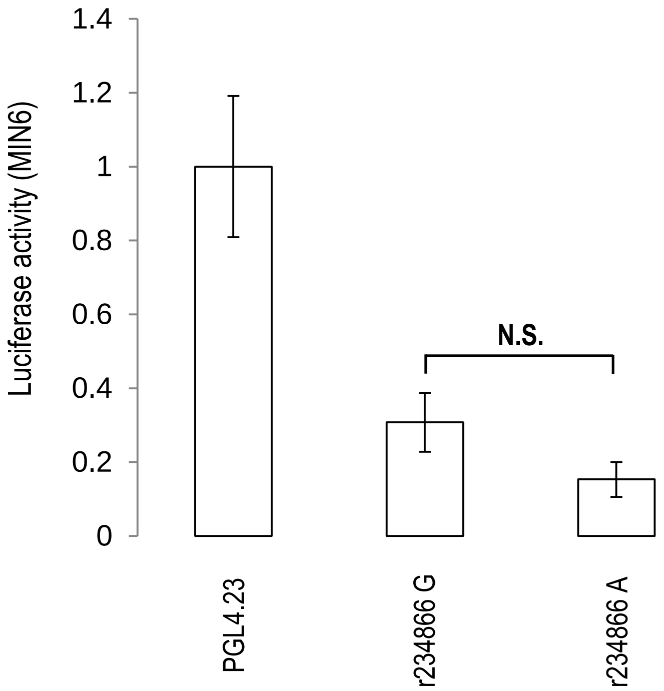

Figure S4. Variant rs234866 at the *KCNQ1* locus overlaps a pancreatic islet predicted enhancer. At the *KCNQ1* association signal indexed by rs233448, the variant rs234866 overlaps a human pancreatic islet predicted enhancer. Luciferase assay for this region did not show enhancer activity for either genotype in MIN6 β-cells. The data are presented as mean ± standard deviation. Three independent experiments were performed in triplicate, and *p*-values were calculated by a two-sided Student's *t*-test.

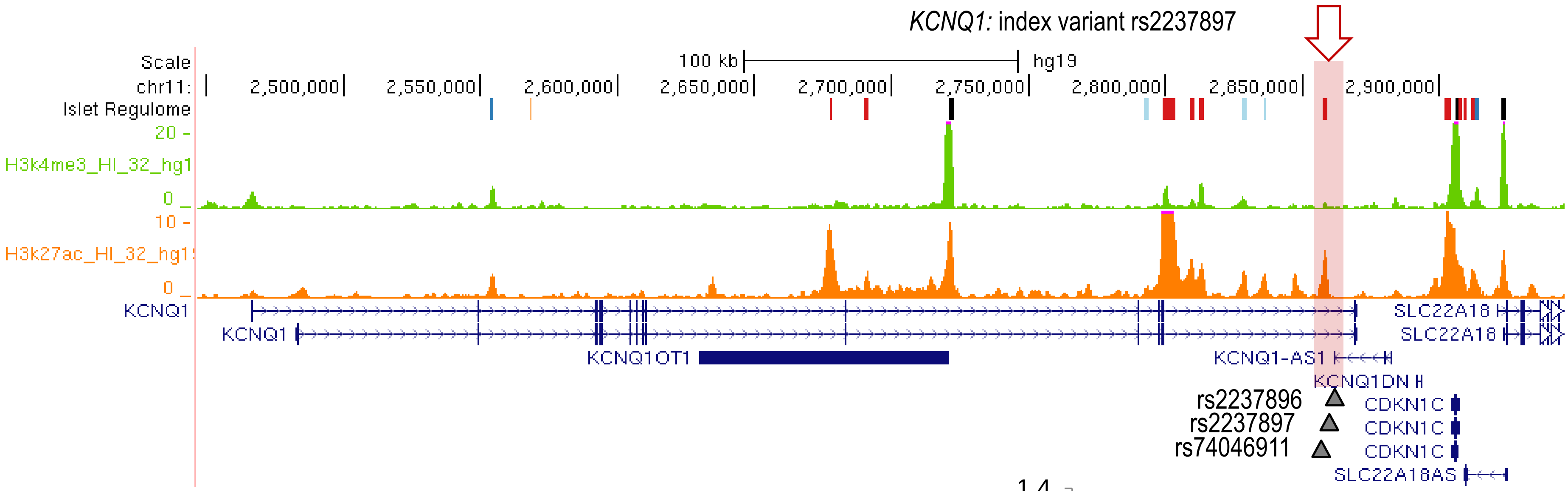

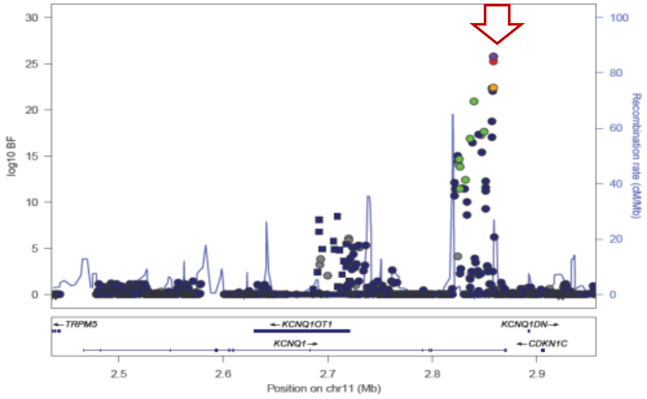

Variant	*Ref*		*Alt*
rs2237896	G	G	A
rs2237897	C	T	T
rs74046911	C	T	T
Frequency	0.83	0.02	0.14

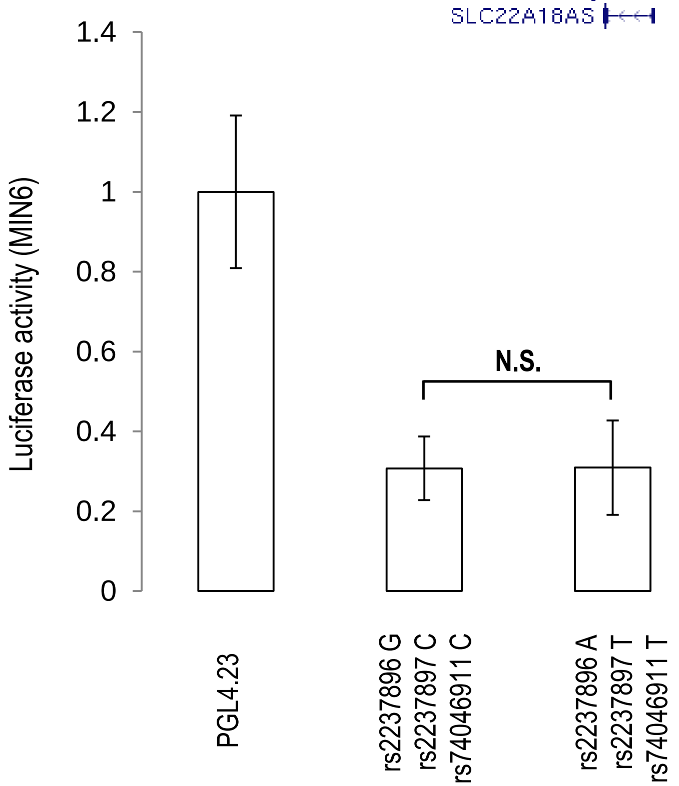

Figure S5. Variant rs2237897 at the *KCNQ1* locus overlaps a pancreatic islet predicted enhancer. At the *KCNQ1* association indexed by rs9368222, variants rs2237896, rs2237897 and rs74046911 overlap one human pancreatic islet predicted enhancer. Luciferase assay did not show enhancer activity in MIN6 β-cells for this region, and we could detect an allele specific difference for the reference vs. alternate haplotypes. The data are presented as mean ± standard deviation. Three independent experiments were performed in triplicate, and *p*-values were calculated by a two-sided Student's *t*-test.

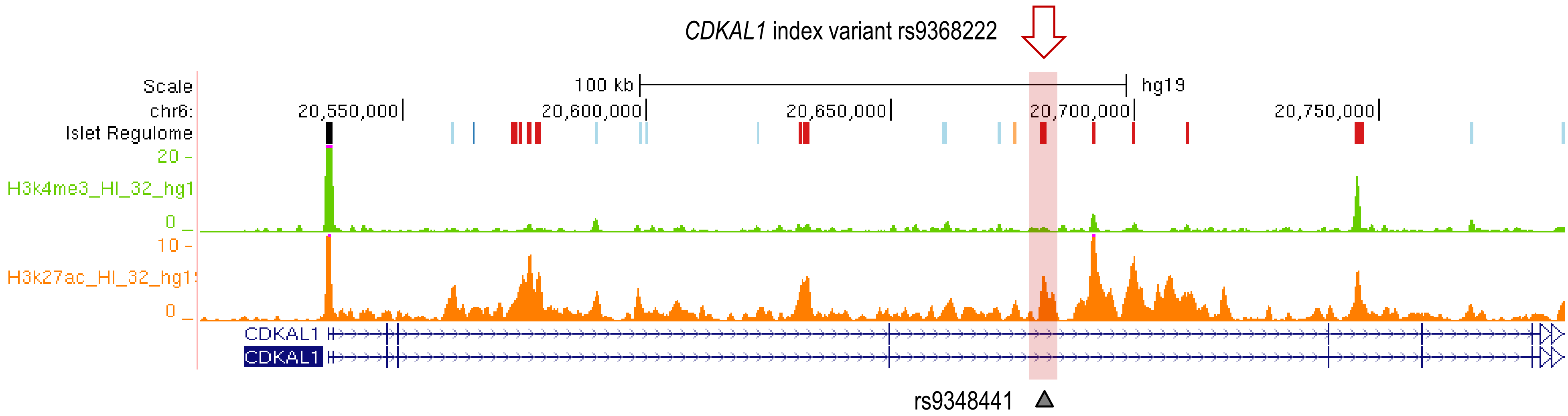

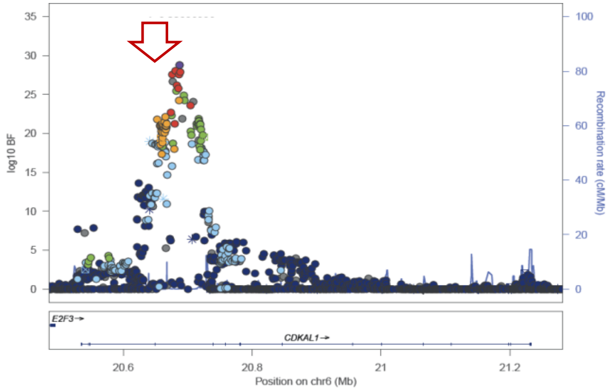

Variant	*Ref*	*Alt*
rs9348441	A	T
Frequency	0.31	0.69

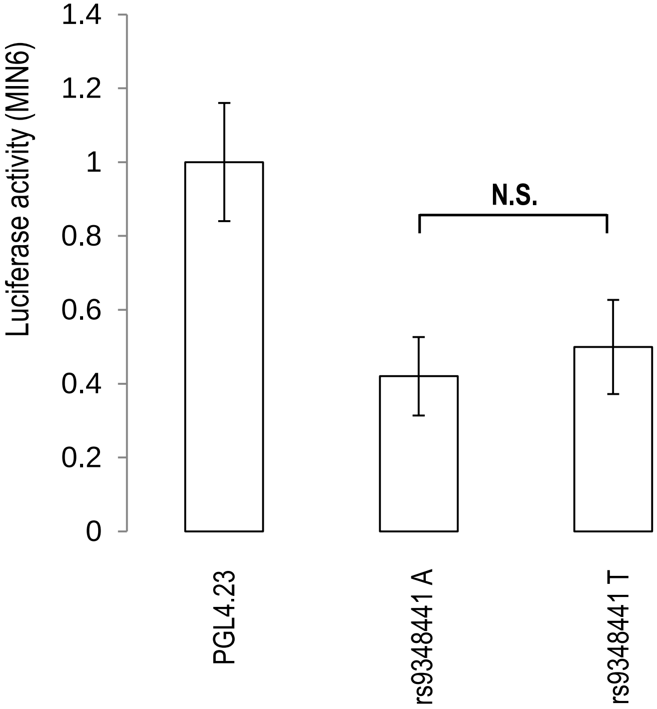

Figure S6. Variant rs9348441 at the *CDKAL1* locus overlaps a pancreatic islet predicted enhancer. At the *CDKAL1* association signal indexed by rs9368222, the variant rs9348441 overlaps a human pancreatic islet predicted enhancer. Luciferase assay for this region did not show enhancer activity for either genotype in MIN6 β-cells. The data are presented as mean ± standard deviation. Three independent experiments were performed in triplicate, and *p*-values were calculated by a two-sided Student's *t*-test.

Table S1. Study sample characteristics and genotyping.

Study	Ancestry group (country of origin)	Case-control status	Sample characteristics					Genotyping array
			Sample size (males/females)	Age (years) mean (SD)	Age at onset (years) mean (SD)	Fasting glucose (mmol/l) mean (SD)	BMI (kg/m2) mean (SD)	
DGDG	European: French<br>(France)	Cases	679 (413/266)	59.5 (10.1)	45.1 (8.4)	9.2 (3.1)	25.9 (2.8)	Affymetrix Human SNP Array 6.0
		Controls	697 (281/416)	53.9 (5.6)		5.1 (0.4)	23.2 (1.8)	
FHS	European<br>(USA)	Cases	677 (386/287)	63.7 (12.4)	N/A	8.6 (2.8)	31.4 (6.5)	Affymetrix Human SNP Array 5.0<br>MIPS 50K
		Controls	7,660 (3,441/4,219)	52.3 (16.0)		5.3 (0.5)	27.0 (5.1)	
FUSION	European: Finnish<br>(Finland)	Cases	1,160 (653/507)	62.9 (7.6)	53.7 (9.1)	9.4 (3.1)	30.2 (4.7)	Illumina Human 300K
		Controls	1,172 (572/600)	63.6 (7.4)		5.3 (0.5)	27.1 (3.9)	
LONGENITY	European: Ashkenazim<br>(USA)	Cases	119 (45/74)	89.6 (13.5)	N/A	N/A	N/A	Affymetrix Human SNP Array 6.0
		Controls	465 (147/318)	85.2 (15.2)		N/A	N/A	
WTCCC	European: UK<br>(UK)	Cases	1,924 (1,118/806)	58.6 (9.2)	50.3 (9.2)	N/A	30.7 (6.1)	Affymetrix Human SNP Array 5.0
		Controls	2,938 (1,446/1,492)	N/A		N/A	N/A	
Starr County	Mexican American<br>(USA)	Cases	837 (333/504)	56.5 (11.8)	46.7 (10.9)	10.0 (4.1)	31.8 (6.4)	Affymetrix Human SNP Array 6.0
		Controls	436 (137/299)	37.6 (9.0)		4.7 (0.5)	29.5 (6.5)	
INDICO	South Asian: Indian<br>(North India)	Cases	1,126 (652/474)	53.43(10.67)	46.02(10.39)	8.59(3.36)	25.27(4.21)	Illumina Human 610K
		Controls	1,135 (597/538)	52.35(10.13)		4.76(0.66)	24.45(4.78)	
LOLIPOP	South Asian: Indian<br>(UK)	Cases	1,783 (1,478/305)	59.4 (9.2)	N/A	8.6 (3.1)	28.1 (4.6)	Illumina Human 610K
		Controls	4,773 (4,048/725)	53.9 (10.7)		5.2 (0.6)	26.8 (4.2)	
PROMIS	South Asian: Pakistani<br>(Pakistan)	Cases	2,310 (1,765/545)	55.0 (9.3)	N/A	N/A	26.0 (4.0)	Illumina Human 660K
		Controls	6,698 (5,561/1,137)	52.9 (10.5)		N/A	25.3 (3.9)	
SINDI	South Asian: Indian<br>(Singapore)	Cases	977 (531/446)	60.7 (9.9)	N/A	9.7 (4.4)	27.1 (5.1)	Illumina Human 610K
		Controls	1,169 (566/603)	55.7 (9.7)		5.4 (1.1)	25.3 (4.4)	
BBJ	East Asian: Japanese<br>(Japan)	Cases	4,470 (3,027/1,429)	65.8 (10.0)	N/A	N/A	25.3 (3.3)	Illumina Human 550K/610K
		Controls	3,071 (1,600/1,300)	52.1 (15.0)		N/A	24.1 (3.0)	
CAGE	East Asian: Japanese<br>(Japan)	Cases	931 (623/308)	66.1 (9.5)	N/A	N/A	24.4 (3.4)	Illumina Human 550K/610K
		Controls	1,404 (844/560)	65.9 (7.4)		N/A	23.1 (3.0)	
CLHNS	East Asian: Filipino<br>(Philippines)	Cases	158 (0/158)	49.6 (6.1)	N/A	N/A	24.4 (3.4)	Affymetrix Human SNP Array 5.0
		Controls	1,523 (0/1,523)	48.3 (6.1)		N/A	23.1 (3.0)	
Hong Kong	East Asian: Chinese<br>(Hong Kong)	Cases	462 (222/240)	56.9 (13.0)	47.5 (13.9)	9.6 (3.7)[a]	25.8 (5.3)	Illumina Human 610K
		Controls	744 (352/392)	37.2 (16.3)		4.7 (0.4)[a]	20.8 (2.0)	
KARE	East Asian: Korean<br>(Korea)	Cases	1,042 (539/503)	56.4 (8.6)	N/A	7.0 (2.6)	25.5 (3.3)	Affymetrix Human SNP Array 5.0
		Controls	2,943 (1,355/1,588)	51.5 (8.6)		4.5 (0.4)	24.1 (3.0)	
SDCS/SP2(1)	East Asian: Chinese<br>(Singapore)	Cases	1,082 (402/680)	65.1 (9.7)	55.7 (12.0)	N/A	25.3 (3.9)	Illumina Human 610K
		Controls	1,006 (217/789)	47.7 (11.1)		4.7 (0.5)	22.3 (3.7)	
SDCS/SP2(2)	East Asian: Chinese<br>(Singapore)	Cases	928 (602/326)	63.7 (10.8)	52.2 (14.4)	N/A	25.4 (3.8)	Illumina Human 1M
		Controls	939 (599/340)	46.7 (10.2)		4.7 (0.5)	22.8 (3.4)	
SiMES	East Asian: Malay<br>(Singapore)	Cases	794 (388/406)	62.3 (9.9)	54.4 (11.2)	N/A	27.8 (4.9)	Illumina Human 610K
		Controls	1,240 (595/645)	56.9 (11.4)		N/A	25.1 (4.8)	
JHS	African American<br>(USA)	Cases	631 (212/419)	59.4 (10.5)	49.9 (11.7)	7.7 (3.1)	34.2 (7.1)	Affymetrix Human SNP Array 6.0
		Controls	2,526 (980/1,546)	53.6 (13.1)		5.0 (0.5)	31.3 (7.3)	

[a]Fasting glucose data obtained from 275 cases and 94 controls.

Table S2. Summary of study-specific quality control, imputation and analysis.

Study	Ancestry group (country of origin)	Sample QC		SNP QC (prior to imputation)			Imputation software	Association analysis	
		Call rate	Relateds	Call rate	HWE	MAF		Software	Covariates
DGDG	European: French (France)	≥0.95	Excluded	≥0.95	$p>10^{-4}$	≥0.01	MACH/minimac	MACH2DAT	Age, sex, BMI, and PCs
FHS	European (USA)	≥0.95	Retained	≥0.95	$p>10^{-6}$	≥0.01	MACH/minimac	R (GEE correction for relatedness)	Age, sex, and PCs
FUSION	European: Finnish (Finland)	≥0.975	Excluded	≥0.90	$p>10^{-6}$	≥0.01	MACH/minimac	MACH2DAT & PLINK	Age, sex, and birth place
LONGENITY	European: Ashkenazim (USA)	≥0.95	Excluded	≥0.95	$p>10^{-3}$	≥0.01	IMPUTE2	SNPTEST2	None
WTCCC	European: UK (UK)	≥0.97	Excluded	≥0.95 (≥0.99 for MAF<0.05)	$p>10^{-3}$	≥0.01	MACH/minimac	MACH2DAT	None
Starr County	Mexican American (USA)	≥0.90	Excluded	≥0.90	None	≥0.01	MACH/minimac	MACH2DAT	Age at diagnosis or enrolment and sex
INDICO	South Asian: Indian (North India)	≥0.95	Excluded	≥0.95 (≥0.99 for MAF<0.05)	$p>5.7\times10^{-7}$ ($p>10^{-4}$ for MAF<0.05)	≥0.01	MACH/minimac	MACH2DAT	Age, sex, BMI, and PCs
LOLIPOP	South Asian: Indian (UK)	≥0.95	Excluded	≥0.95	$p>10^{-6}$	≥0.01	MACH/minimac	MACH2DAT	Age, sex, CHD, cohort, and PCs
PROMIS	South Asian: Pakistani (Pakistan)	≥0.95	Excluded	≥0.97	$p>10^{-6}$	≥0.01	MACH/minimac	MACH2DAT & PLINK	PCs
SINDI	South Asian: Indian (Singapore)	≥0.95	Excluded	≥0.97	$p>10^{-6}$	≥0.01	MACH/minimac	MACH2DAT	None
BBJ	East Asian: Japanese (Japan)	≥0.98	Excluded	≥0.99	$p>10^{-6}$	≥0.01	MACH/minimac	MACH2DAT	None
CAGE	East Asian: Japanese (Japan)	≥0.90	Excluded	≥0.95	$p>10^{-6}$	≥0.01	MACH/minimac	MACH2DAT	None
CLHNS	East Asian: Filipino (Philippines)	≥0.97	Excluded	≥0.90	$p>10^{-6}$	≥0.01	MACH/minimac	MACH2DAT	Age, BMI, and household assets
Hong Kong	East Asian: Chinese (Hong Kong)	≥0.98	Excluded	≥0.95 (≥0.99 for MAF<0.05)	$p>10^{-4}$	≥0.01	MACH/minimac	MACH2DAT	Age and sex
KARE	East Asian: Korean (Korea)	≥0.98	Excluded	≥0.90	$p>10^{-6}$	≥0.01	MACH/minimac	MACH2DAT	Age, sex and recruitment area
SDCS/SP2(1)	East Asian: Chinese (Singapore)	≥0.95	Excluded	≥0.95	$p>10^{-4}$	≥0.01	MACH/minimac	MACH2DAT	None
SDCS/SP2(2)	East Asian: Chinese (Singapore)	≥0.95	Excluded	≥0.95	$p>10^{-4}$	≥0.01	MACH/minimac	MACH2DAT	None
SiMES	East Asian: Malay (Singapore)	≥0.95	Excluded	≥0.95	$p>10^{-6}$	≥0.01	MACH/minimac	MACH2DAT	None
JHS	African American (USA)	≥0.95	Retained	≥0.90	None	≥0.01	MACH/minimac	GWAF (LMM to account for relatedness)	PCs

Table S3. Summary statistics from the transancestral meta-analysis (22,086 cases and 42,539 controls) for distinct association signals at each locus, sub-divided by ancestry group and ancestral clade.

***IGF2BP2*: rs11705729; risk allele T, other allele C. Unconditional meta-analysis.**

Ancestry group	Risk allele frequency	Sample size		MANTRA		Fixed-effects meta-analysis		
		Cases	Controls	$Log_{10}BF$	$Log_{10}BF$ heterogeneity	OR (95% CI)	*p*-value	Cochran's *Q* *p*-value
South Asian	0.44	6,196	13,775	4.77	-0.03	1.12 (1.07-1.17)	$7.7x10^{-7}$	0.82
European	0.33	4,555	12,932	4.54	0.01	1.15 (1.09-1.22)	$2.0x10^{-6}$	0.13
Mexican American	0.27	837	436	-0.34	N/A	1.23 (1.00-1.50)	0.050	N/A
Eur-MexAm-SAsia clade	0.39	11,588	27,143	10.23	-0.17	1.13 (1.09-1.17)	$1.8x10^{-12}$	0.42
East Asian	0.31	9,867	12,870	8.06	-0.04	1.15 (1.10-1.20)	$3.8x10^{-10}$	0.32
African American	0.79	631	2,526	0.12	N/A	1.13 (0.97-1.32)	0.11	N/A
**Combined**		**22,086**	**42,539**	**19.35**	**-0.05**	**1.14 (1.11-1.17)**	$\mathbf{1.3x10^{-21}}$	**0.49**

***CDKAL1*: rs9368222; risk allele A, other allele C. Unconditional meta-analysis.**

Ancestry group	Risk allele frequency	Sample size		MANTRA		Fixed-effects meta-analysis		
		Cases	Controls	$Log_{10}BF$	$Log_{10}BF$ heterogeneity	OR (95% CI)	*p*-value	Cochran's *Q* *p*-value
South Asian	0.24	6,196	13,775	1.30	-0.01	1.08 (1.02-1.13)	0.0043	0.28
European	0.28	4,555	12,932	9.64	-0.07	1.23 (1.16-1.31)	$7.8x10^{-12}$	0.33
Mexican American	0.31	837	436	-0.19	N/A	1.11 (0.91-1.35)	0.32	N/A
Eur-MexAm-SAsia clade	0.26	11,588	27,143	10.28	0.99	1.14 (1.10-1.18)	$2.3x10^{-11}$	0.02
East Asian	0.42	9,867	12,870	18.65	0.06	1.21 (1.16-1.26)	$8.4x10^{-21}$	0.09
African American	0.17	631	2,526	0.17	N/A	1.14 (0.97-1.36)	0.12	N/A
**Combined**		**22,086**	**42,539**	**28.84**	**0.99**	**1.17 (1.14-1.21)**	$\mathbf{4.1x10^{-30}}$	**0.0058**

***CDKN2A-B*: rs10965246; risk allele T, other allele C. Conditional meta-analysis adjusting for rs10757282.**

Ancestry group	Risk allele frequency	Sample size		MANTRA		Fixed-effects meta-analysis		
		Cases	Controls	$Log_{10}BF$	$Log_{10}BF$ heterogeneity	OR (95% CI)	*p*-value	Cochran's *Q* *p*-value
South Asian	0.87	6,196	13,775	10.16	0.09	1.33 (1.23-1.44)	$3.1x10^{-12}$	0.089
European	0.83	4,555	12,932	9.70	0.17	1.35 (1.24-1.47)	$1.1x10^{-11}$	0.0081
Mexican American	0.88	837	436	0.05	N/A	1.33 (0.99-1.80)	0.067	N/A
Eur-MexAm-SAsia clade	0.85	11,588	27,143	37.45	-0.03	1.34 (1.26-1.42)	$4.0x10^{-23}$	0.016
East Asian	0.59	9,867	12,870	16.25	0.17	1.28 (1.21-1.36)	$2.6x10^{-18}$	0.018
African American	0.92	631	2,526	-0.05	N/A	1.18 (0.92-1.51)	0.20	N/A
**Combined**		**22,086**	**42,539**	**37.45**	**-0.03**	**1.31 (1.26-1.36)**	$\mathbf{8.4x10^{-40}}$	**0.0029**

***CDKN2A-B*: rs10757282; risk allele C, other allele T. Conditional meta-analysis adjusting for rs10965246.**

**Ancestry group**	**Risk allele frequency**	**Sample size**		**MANTRA**		**Fixed-effects meta-analysis**		
		**Cases**	**Controls**	**$Log_{10}BF$**	**$Log_{10}BF$ heterogeneity**	**OR (95% CI)**	***p*-value**	**Cochran's *Q* *p*-value**
South Asian	0.42	6,192	13,770	3.20	0.60	1.12 (1.06-1.17)	$3.0x10^{-5}$	0.79
European	0.42	4,555	12,932	5.04	0.48	1.20 (1.11-1.28)	$7.4x10^{-7}$	0.026
Mexican American	0.47	837	436	-0.61	N/A	1.04 (0.86-1.27)	0.68	N/A
Eur-MexAm-SAsia clade	0.42	11,584	27,138	6.68	-1.32	1.14 (1.09-1.19)	$4.4x10^{-10}$	0.083
East Asian	0.61	9,861	12,868	2.34	0.00	1.11 (1.05-1.17)	0.00030	0.57
African American	0.25	631	2,526	-0.46	N/A	1.01 (0.87-1.17)	0.87	N/A
**Combined**		**22,081**	**42,532**	**10.31**	**0.01**	**1.12 (1.09-1.16)**	**$2.0x10^{-12}$**	**0.17**

***KCNQ1*: rs231353; risk allele G, other allele A. Conditional meta-analysis adjusting for rs234864 and rs2237896.**

**Ancestry group**	**Risk allele frequency**	**Sample size**		**MANTRA**		**Fixed-effects meta-analysis**		
		**Cases**	**Controls**	**$Log_{10}BF$**	**$Log_{10}BF$ heterogeneity**	**OR (95% CI)**	***p*-value**	**Cochran's *Q* *p*-value**
South Asian	0.60	6,196	13,775	3.88	0.05	1.11 (1.06-1.17)	$8.3x10^{-6}$	0.42
European	0.37	4,555	12,932	1.75	0.15	1.10 (1.04-1.17)	0.0013	0.34
Mexican American	0.52	837	436	0.34	N/A	1.20 (1.00-1.44)	0.056	N/A
Eur-MexAm-SAsia clade	0.49	11,588	27,143	6.70	-0.08	1.11 (1.07-1.15)	$8.4x10^{-9}$	0.53
East Asian	0.82	9,867	12,870	2.00	-0.06	1.10 (1.04-1.15)	0.00069	0.71
African American	0.45	631	2,526	-0.18	N/A	0.93 (0.82-1.06)	0.30	N/A
**Combined**		**22,086**	**42,539**	**9.29**	**-0.13**	**1.11 (1.07-1.14)**	**$1.7x10^{-11}$**	**0.79**

***KCNQ1*: rs233448; risk allele C, other allele T. Conditional meta-analysis adjusting for rs231353 and rs2237896.**

**Ancestry group**	**Risk allele frequency**	**Sample size**		**MANTRA**		**Fixed-effects meta-analysis**		
		**Cases**	**Controls**	**$Log_{10}BF$**	**$Log_{10}BF$ heterogeneity**	**OR (95% CI)**	***p*-value**	**Cochran's *Q* *p*-value**
South Asian	0.78	6,156	13,693	3.75	0.08	1.13 (1.07-1.19)	$6.0x10^{-6}$	0.60
European	0.73	4,555	12,932	2.05	-0.11	1.13 (1.05-1.20)	0.00055	0.34
Mexican American	0.78	837	436	-0.21	N/A	1.16 (0.91-1.48)	0.22	N/A
Eur-MexAm-SAsia clade	0.75	11,548	27,061	6.93	0.13	1.13 (1.08-1.18)	$6.0x10^{-9}$	0.69
East Asian	0.86	9,867	12,870	2.64	0.12	1.13 (1.07-1.21)	$7.6x10^{-5}$	0.061
African American	0.85	631	2,526	-0.36	N/A	0.95 (0.78-1.14)	0.55	N/A
**Combined**		**22,046**	**42,457**	**9.65**	**0.14**	**1.12 (1.09-1.16)**	**$9.5x10^{-12}$**	**0.18**

***KCNQ1*: rs2237897; risk allele C, other allele T. Conditional meta-analysis adjusting for rs231353 and rs234864.**

**Ancestry group**	**Risk allele frequency**	**Sample size**		**MANTRA**		**Fixed-effects meta-analysis**		
		**Cases**	**Controls**	**$Log_{10}BF$**	**$Log_{10}BF$ heterogeneity**	**OR (95% CI)**	***p*-value**	**Cochran's *Q* *p*-value**
South Asian	0.98	6,196	13,775	1.13	0.09	1.29 (1.06-1.57)	0.010	0.41
European	0.93	4,555	12,932	1.55	0.23	1.29 (1.10-1.51)	0.0018	0.67
Mexican American	0.80	837	436	0.59	N/A	1.37 (1.04-1.81)	0.027	N/A
Eur-MexAm-SAsia clade	0.95	11,588	27,143	4.04	-0.07	1.30 (1.16-1.45)	4.4×10^{-6}	0.80
East Asian	0.65	9,867	12,870	5.55	0.29	1.17 (1.10-1.24)	2.0×10^{-7}	0.12
African American	0.92	631	2,526	-0.14	N/A	1.18 (0.91-1.52)	0.22	N/A
**Combined**		**22,086**	**42,539**	**9.79**	**0.17**	**1.19 (1.14-1.26)**	$\mathbf{7.7 \times 10^{-12}}$	**0.35**

OR: odds-ratio. CI: confidence interval. BF: Bayes' factor.

Table S4. Membership of 99% credible sets after transancestral meta-analysis (22,086 cases and 42,539 controls).

Locus	Index variant	99% credible set variants								
		Variant	Chr	Position (b37)	Risk allele	Other allele	*p*-value	OR (95% CI)	Posterior probability of driving association signal (π_C)	Mean (range) r^2 or info
IGF2BP2	rs11705729	rs11705729	3	185,507,299	T	A	$1.3x10^{-21}$	1.14 (1.11-1.17)	0.128	0.96 (0.74-1.00)
		rs6765808	3	185,508,029	T	C	$1.0x10^{-21}$	1.14 (1.11-1.17)	0.108	0.97 (0.75-1.00)
		rs9841201	3	185,505,984	A	G	$1.5x10^{-21}$	1.14 (1.11-1.17)	0.101	0.97 (0.74-1.00)
		rs4389513	3	185,506,857	G	T	$1.8x10^{-21}$	1.14 (1.11-1.17)	0.101	0.97 (0.74-1.00)
		rs7633675	3	185,510,613	G	T	$2.5x10^{-21}$	1.14 (1.11-1.17)	0.061	0.98 (0.79-1.00)
		rs34782298	3	185,520,996	A	G	$3.9x10^{-21}$	1.14 (1.11-1.17)	0.047	0.94 (0.75-0.98)
		rs6769511	3	185,530,290	C	T	$4.8x10^{-21}$	1.13 (1.11-1.17)	0.039	0.99 (0.83-1.00)
		rs13100823	3	185,514,088	T	C	$5.3x10^{-21}$	1.14 (1.11-1.17)	0.038	0.97 (0.75-1.00)
		rs7615045	3	185,521,126	G	A	$4.3x10^{-21}$	1.13 (1.10-1.16)	0.031	0.98 (0.80-1.00)
		rs73063004	3	185,526,345	T	C	$4.2x10^{-21}$	1.13 (1.10-1.16)	0.030	0.99 (0.83-1.00)
		rs7646519	3	185,523,853	A	G	$5.0x10^{-21}$	1.14 (1.11-1.17)	0.030	0.97 (0.80-1.00)
		rs11927381	3	185,508,591	C	T	$1.8x10^{-21}$	1.14 (1.11-1.17)	0.028	0.97 (0.75-1.00)
		rs1470580	3	185,529,174	A	T	$4.9x10^{-21}$	1.13 (1.11-1.17)	0.027	0.99 (0.83-1.00)
		rs9859406	3	185,534,482	A	G	$6.2x10^{-21}$	1.14 (1.11-1.17)	0.026	0.97 (0.79-1.00)
		rs9808924	3	185,514,189	A	G	$6.4x10^{-21}$	1.14 (1.11-1.17)	0.022	0.96 (0.75-0.99)
		rs7631557	3	185,513,646	C	G	$8.2x10^{-21}$	1.14 (1.11-1.17)	0.021	0.98 (0.75-1.00)
		rs73061095	3	185,516,807	G	T	$8.2x10^{-21}$	1.13 (1.10-1.16)	0.017	0.98 (0.76-1.00)
		rs7619401	3	185,510,006	A	G	$1.4x10^{-20}$	1.13 (1.10-1.16)	0.013	0.98 (0.79-1.00)
		rs7640539	3	185,513,296	A	T	$1.7x10^{-20}$	1.13 (1.10-1.16)	0.012	0.98 (0.77-1.00)
		rs11929397	3	185,510,190	C	T	$1.5x10^{-20}$	1.13 (1.10-1.16)	0.011	0.98 (0.80-1.00)
		rs11716713	3	185,515,610	C	G	$1.7x10^{-20}$	1.13 (1.10-1.16)	0.010	0.98 (0.76-1.00)
		rs4481184	3	185,505,787	T	C	$1.6x10^{-20}$	1.13 (1.10-1.17)	0.010	0.97 (0.76-1.00)
		rs4686696	3	185,516,520	A	G	$1.7x10^{-20}$	1.13 (1.10-1.16)	0.009	0.98 (0.76-1.00)
		rs11928319	3	185,518,241	C	G	$2.1x10^{-20}$	1.13 (1.10-1.16)	0.008	0.98 (0.77-1.00)
		rs6786649	3	185,514,223	A	C	$1.6x10^{-20}$	1.13 (1.10-1.16)	0.008	0.98 (0.76-1.00)
		rs71320321	3	185,519,107	A	G	$2.8x10^{-20}$	1.13 (1.10-1.16)	0.007	0.98 (0.77-1.00)
		rs4414887	3	185,506,892	T	C	$2.2x10^{-20}$	1.13 (1.10-1.16)	0.006	0.98 (0.76-1.00)
		rs6767484	3	185,520,578	G	A	$3.0x10^{-20}$	1.13 (1.10-1.16)	0.006	0.98 (0.78-1.00)
		rs7646518	3	185,514,931	C	T	$1.4x10^{-20}$	1.13 (1.10-1.16)	0.006	0.98 (0.76-1.00)
		rs9854769	3	185,520,948	G	A	$3.1x10^{-20}$	1.13 (1.10-1.16)	0.005	0.97 (0.79-1.00)
		rs7633811	3	185,519,848	G	C	$3.3x10^{-20}$	1.13 (1.10-1.16)	0.005	0.98 (0.78-1.00)
		rs7637773	3	185,515,635	A	G	$1.9x10^{-20}$	1.13 (1.10-1.16)	0.004	0.98 (0.76-1.00)
		rs150111048	3	185,514,421	G	A	$4.3x10^{-20}$	1.15 (1.12-1.19)	0.004	0.79 (0.56-0.88)
		rs6780171	3	185,503,456	A	T	$2.1x10^{-20}$	1.13 (1.10-1.17)	0.004	0.96 (0.71-0.99)
		rs76922886	3	185,518,921	A	G	$3.8x10^{-20}$	1.13 (1.10-1.16)	0.004	0.98 (0.76-1.00)
		rs10428126	3	185,520,342	C	T	$4.5x10^{-20}$	1.13 (1.10-1.16)	0.004	0.99 (0.79-1.00)

CDKAL1	rs9368222	rs9368222	6	20,686,996	A	C	4.1×10^{-30}	1.17 (1.14-1.21)	0.711	0.97 (0.74-1.00)
		rs9348441	6	20,680,678	A	T	2.3×10^{-27}	1.16 (1.13-1.20)	0.120	0.97 (0.75-1.00)
		rs10440833	6	20,688,121	A	T	4.5×10^{-27}	1.16 (1.13-1.20)	0.081	0.95 (0.69-0.99)
		rs35261542	6	20,675,792	A	C	5.4×10^{-28}	1.17 (1.13-1.20)	0.042	0.95 (0.70-1.00)
		rs7766070	6	20,686,573	A	C	2.5×10^{-28}	1.17 (1.13-1.20)	0.038	0.96 (0.74-1.00)
CDKN2A-B	rs10965246	rs10965246	9	22,132,698	T	C	8.4×10^{-40}	1.31 (1.26-1.36)	0.526	0.94 (0.79-1.00)
		rs10965248	9	22,132,878	T	C	7.2×10^{-40}	1.31 (1.26-1.36)	0.313	0.94 (0.78-1.00)
		rs10965247	9	22,132,729	A	G	4.1×10^{-39}	1.30 (1.25-1.36)	0.090	0.94 (0.79-0.99)
		rs10965250	9	22,133,284	G	A	1.1×10^{-38}	1.31 (1.26-1.36)	0.051	0.93 (0.78-1.00)
		rs10811660	9	22,134,068	G	A	2.8×10^{-38}	1.31 (1.26-1.36)	0.018	0.93 (0.76-1.00)
CDKN2A-B	rs10757282	rs10757282	9	22,133,984	C	T	2.0×10^{-12}	1.12 (1.09-1.16)	0.311	0.92 (0.32-1.00)
		rs7019778	9	22,134,651	C	A	2.8×10^{-12}	1.12 (1.09-1.16)	0.250	0.90 (0.32-0.99)
		rs7019437	9	22,134,302	G	C	3.2×10^{-12}	1.12 (1.09-1.16)	0.136	0.88 (0.33-0.99)
		rs10217762	9	22,133,645	C	T	6.9×10^{-12}	1.12 (1.09-1.16)	0.122	0.91 (0.33-1.00)
		rs10757283	9	22,134,172	T	C	7.6×10^{-12}	1.12 (1.08-1.15)	0.086	0.90 (0.32-1.00)
		rs7018475	9	22,137,685	G	T	6.0×10^{-12}	1.13 (1.09-1.16)	0.084	0.67 (0.45-1.00)
		rs7045889	9	22,133,251	A	G	5.4×10^{-11}	1.12 (1.08-1.16)	0.009	0.83 (0.53-1.00)
KCNQ1	rs231353	rs231353	11	2,709,019	G	A	1.7×10^{-11}	1.11 (1.07-1.14)	0.637	0.93 (0.68-0.99)
		rs231361	11	2,691,500	A	G	3.2×10^{-11}	1.10 (1.07-1.14)	0.346	0.92 (0.69-1.00)
		rs231362	11	2,691,471	G	A	1.7×10^{-9}	1.11 (1.07-1.15)	0.013	0.87 (0.58-1.00)
KCNQ1	rs233448	rs233448	11	2,840,424	C	T	9.5×10^{-12}	1.12 (1.09-1.16)	0.602	0.94 (0.84-1.00)
		rs151293	11	2,841,365	C	A	4.5×10^{-11}	1.12 (1.08-1.16)	0.141	0.95 (0.84-1.00)
		rs233449	11	2,843,803	G	A	8.1×10^{-11}	1.12 (1.08-1.15)	0.090	0.87 (0.70-1.00)
		rs234866	11	2,857,897	G	A	1.2×10^{-10}	1.13 (1.09-1.17)	0.048	0.77 (0.55-0.87)
		rs233450	11	2,844,048	A	G	2.5×10^{-10}	1.11 (1.08-1.15)	0.034	0.88 (0.72-1.00)
		rs234856	11	2,852,328	G	T	8.3×10^{-11}	1.12 (1.08-1.16)	0.034	0.91 (0.71-0.99)
		rs234858	11	2,852,857	G	A	2.8×10^{-10}	1.11 (1.08-1.15)	0.017	0.90 (0.72-0.98)
		rs233447	11	2,837,723	C	T	7.2×10^{-10}	1.11 (1.07-1.15)	0.008	0.94 (0.74-0.99)
		rs233446	11	2,837,625	A	C	1.5×10^{-9}	1.11 (1.07-1.14)	0.006	0.93 (0.71-1.00)
		rs233451	11	2,846,427	G	A	1.1×10^{-9}	1.12 (1.08-1.16)	0.005	0.88 (0.68-0.98)
		rs234857	11	2,852,529	T	C	8.0×10^{-10}	1.11 (1.07-1.14)	0.005	0.93 (0.72-1.00)
KCNQ1	rs2237897	rs2237897	11	2,858,546	C	T	7.7×10^{-12}	1.19 (1.14-1.26)	0.392	0.75 (0.35-0.97)
		rs2237896	11	2,858,440	G	A	9.5×10^{-12}	1.19 (1.14-1.26)	0.338	0.79 (0.37-1.00)
		rs74046911	11	2,858,636	C	T	1.3×10^{-11}	1.19 (1.13-1.25)	0.260	0.71 (0.34-0.94)

Chr: chromosome. OR: odds-ratio. CI: confidence interval.

Membership of the T2D-GENES Consortium

Hanna E Abboud[1], Goncalo Abecasis[2], Uzma Afzal[3], Vineeta Agarwala[4,5], David Aguilar[6], David Altshuler[5,7,8,9,10,11], Rector Arya[12], Jennifer L Asimit[13], Gil Atzmon[14,15], Tin Aung[16,17,18], Ines Barroso[13,19], Nir Barzilai[14], Nicola L Beer[20], Graeme I Bell[21], Jennifer E Below[22], Dwaipayan Bharadwaj[23], John Blangero[24], Michael Boehnke[2], Donald W Bowden[25,26,27], Nöel Burtt[5], John C Chambers[3,28,29], Edmund Chan[30], Juliana CN Chan[31,32,33], Giriraj R Chandak[34], Han Chen[35,36], Peng Chen[37], Ching-Yu Cheng[16,17,18,37], Kee Seng Chia[37], Yoon Shin Cho[38], Pablo Cingolani[39,40], Adolfo Correa[41], Nancy J Cox[42], Joanne E Curran[24], Aaron G Day-Williams[13], Ralph A DeFronzo[1], Peter Donnelly[43,44], Ravindranath Duggirala[45], Josee Dupuis[35,46], Shah B Ebrahim[47], Paul Elliott[3,48], Vidya S Farook[45], Juan Fernandez Tajes[43], Teresa Ferreira[43], Tasha Fingerlin[49], Jason Flannick[5,9], Jose C Florez[5,8,10,50], Pierre Fontanillas[5], Sharon P Fowler[1], Timothy M Frayling[51], Barry I Freedman[52], Philippe Froguel[53], Christian Fuchsberger[2], Eric R Gamazon[42], Kyle J Gaulton[43], Benjamin Glaser[54], Anna L Gloyn[20,43,55], Min Jin Go[56], Michael Griswold[57], Daniel Esten Hale[12], Bok-Ghee Han[56], Craig L Hanis[22], Andrew T Hattersley[58], Pamela J Hicks[25,26,27], Heather M Highland[59], Momoko Horikoshi[20,43], Cheng Hu[60], Iksoo Huh[61], Jeroen R Huyghe[2], Mohammad Kamran Ikram[16,17,18], Hae Kyung Im[42], Kathleen A Jablonski[62], Anne U Jackson[2], Christopher P Jenkinson[1,63], Weiping Jia[60], Hyun Min Kang[2], Chiea-Chuen Khor[16,17,37,64,65], Bong-Jo Kim[56], Yongkang Kim[61], Young Jin Kim[56], Jaspal Singh Kooner[28,29,66], Satish Kumar[24], Teemu Kuulasmaa[67], Johanna Kuusisto[67,68], Min-Seok Kwon[69], Markku Laakso[67,68], Jaehoon Lee[61], Jong-Young Lee[56], Juyoung Lee[56], Selyeong Lee[61], Donna M Lehman[1], Benjamin Lehne[3], Wei Yen Lim[37], Keng-Han Lin[2], Jianjun Liu[37,65], Adam E Locke[2], Marie Loh[3,70,71], Ronald CW Ma[31,32,33], Anubha Mahajan[43], Alisa Manning[5], Taylor J Maxwell[22], Mark I McCarthy[20,43,55], James B Meigs[72], Karen L Mohlke[73], Andrew P Morris[43,74], Solomon K Musani[75], Yoshihiko Nagai[40,76,77], Maggie CY Ng[26,27], Nicholette D Palmer[25,26,27], Taesung Park[61,69], Toni I Pollin[78], Dorairaj Prabhakaran[47], Sobha Puppala[45], Manuel A Rivas[43], Neil R Robertson[43], Manjinder Sandhu[13,79], James Scott[66], William R Scott[3], Mark Seielstad[80,81], Xueling Sim[2], Rob Sladek[40,76,82], Alena Stančáková[67,83], E Shyong Tai[30,37,84], Sian-Tsung Tan[28,66], Nikhil Tandon[85], Herman A Taylor[41], Yik Ying Teo[37,86,87], Tanya M Teslovich[2], Farook Thameem[1], Xu Wang[37], Richard M Watanabe[88,89,90], Ryan P Welch[2], Gregory Wilson[91], James G Wilson[92], Tien Yin Wong[16,17,18], Joon Yoon[69], Eleftheria Zeggini[13], Weihua Zhang[3,28]

[1]Department of Medicine, University of Texas Health Science Center, San Antonio, Texas, 78229, USA. [2]Department of Biostatistics and Center for Statistical Genetics, University of Michigan, Ann Arbor, Michigan, 48109, USA. [3]Department of Epidemiology and Biostatistics, Imperial College London, London, W2 1PG, UK. [4]Harvard-MIT Division of Health Sciences and Technology, Massachusetts Institute of Technology, Cambridge, Massachusetts, USA. [5]Program in Medical and Population Genetics, Broad Institute, Cambridge, Massachusetts, 02142, USA. [6]Cardiovascular Division, Baylor College of Medicine, Houston, Texas, USA. [7]Department of Genetics, Harvard Medical School, Boston, Massachusetts, 02115, USA. [8]Department of Medicine, Harvard Medical School, Boston, Massachusetts, 02115, USA. [9]Department of Molecular Biology, Massachusetts General Hospital, Boston, Massachusetts, 02114, USA. [10]Diabetes Research Center (Diabetes Unit), Department of Medicine, Massachusetts General Hospital, Boston, Massachusetts, 02114, USA. [11]Department of Biology, Massachusetts Institute of Technology, Cambridge, Massachusetts, 02139, USA. [12]Department of Pediatrics, University of Texas Health Science Center, San Antonio, Texas, 78229, USA. [13]Department of Human Genetics, Wellcome Trust Sanger Institute, Hinxton, Cambridgeshire, CB10 1SA, UK. [14]Departments of Medicine and Genetics, Albert Einstein College of

Medicine, Bronx, New York, 10461, USA. [15]Department of Natural Science, University of Haifa, Haifa, Israel. [16]Singapore Eye Research Institute, Singapore National Eye Centre, 168751, Singapore. [17]Department of Ophthalmology, Yong Loo Lin School of Medicine, National University of Singapore, National University Health System, 119228, Singapore. [18]The Eye Academic Clinical Programme, Duke-NUS Graduate Medical School, 169857, Singapore. [19]Metabolic Research Laboratories, Institute of Metabolic Science, University of Cambridge, Cambridge, CB22 0QQ, UK. [20]Oxford Centre for Diabetes, Endocrinology and Metabolism, Radcliffe Department of Medicine, University of Oxford, Old Road, Headington, Oxford, OX3 7LE, UK. [21]Departments of Medicine and Human Genetics, The University of Chicago, Chicago, Illinois, 60637, USA. [22]Human Genetics Center, School of Public Health, The University of Texas Health Science Center at Houston, Houston, Texas, USA. [23]Functional Genomics Unit, CSIR-Institute of Genomics & Integrative Biology (CSIR-IGIB), New Delhi, 110007, India. [24]South Texas Diabetes and Obesity Institute, Regional Academic Health Center, University of Texas Health Science Center at San Antonio / University of Texas Rio Grande Valley, Brownsville, Texas, 78520, USA. [25]Department of Biochemistry, Wake Forest School of Medicine, Winston-Salem, North Carolina, 27157, USA. [26]Center for Genomics and Personalized Medicine Research, Wake Forest School of Medicine, Winston-Salem, North Carolina, 27157, USA. [27]Center for Diabetes Research, Wake Forest School of Medicine, Winston-Salem, North Carolina, 27157, USA. [28]Department of Cardiology, Ealing Hospital NHS Trust, Southall, Middlesex, UB1 3HW, UK. [29]Imperial College Healthcare NHS Trust, Imperial College London, London, W12 0HS, UK. [30]Department of Medicine, Yong Loo Lin School of Medicine, National University of Singapore, National University Health System, 119228, Singapore. [31]Department of Medicine and Therapeutics, The Chinese University of Hong Kong, Hong Kong, China. [32]Li Ka Shing Institute of Health Sciences, The Chinese University of Hong Kong, Hong Kong, China. [33]Hong Kong Institute of Diabetes and Obesity, The Chinese University of Hong Kong, Hong Kong, China. [34]CSIR-Centre for Cellular and Molecular Biology, Habsiguda, Hyderabad, Telangana, 500007, India. [35]Department of Biostatistics, Boston University School of Public Health, Boston, Massachusetts, 02118, USA. [36]Department of Biostatistics, Harvard School of Public Health, Boston, Massachusetts, 02115, USA. [37]Saw Swee Hock School of Public Health, National University of Singapore, National University Health System, 117597, Singapore. [38]Department of Biomedical Science, Hallym University, Chuncheon, Republic of Korea. [39]School of Computer Science, McGill University, McConnell Engineering Building, Rm 318, Montreal, Quebec, H3A 0E9, Canada. [40]McGill University and Génome Québec Innovation Centre, Montreal, Quebec, H3A 0G1, Canada. [41]Department of Medicine, University of Mississippi Medical Center, Jackson, Mississippi, USA. [42]Department of Medicine, Section of Genetic Medicine, The University of Chicago, Chicago, Illinois, 60637, USA. [43]Wellcome Trust Centre for Human Genetics, Nuffield Department of Medicine, University of Oxford, Oxford, OX3 7BN, UK. [44]Department of Statistics, University of Oxford, Oxford, OX1 3TG, UK. [45]Department of Genetics, Texas Biomedical Research Institute, San Antonio, Texas, 78227, USA. [46]National Heart, Lung, and Blood Institute's Framingham Heart Study, Framingham, Massachusetts, 01702, USA. [47]Centre for Chronic Disease Control, New Delhi, 110016, India. [48]MRC-PHE Centre for Environment and Health, Imperial College London, St Mary's Campus, Norfolk Place, London, W2 1PG, UK. [49]Department of Epidemiology, Colorado School of Public Health, University of Colorado, Building 500, Room W3131, Anschutz Medical Campus, Aurora, Colorado, 80045, USA. [50]Center for Human Genetic Research, Department of Medicine, Massachusetts General Hospital, Boston, Massachusetts, 02114, USA. [51]Genetics of Complex Traits, University of Exeter Medical School, University of Exeter, Exeter, EX1 2LU, UK. [52]Department of Internal Medicine, Section on Nephrology, Wake Forest School of Medicine, Winston-Salem, North Carolina, 27157, USA. [53]Genomics and Molecular Physiology, CNRS (Institut de Biologie de Lille), 2 Rue des Canonniers, Lille, 59046 Cedex, France. [54]Endocrinology and Metabolism Service, Hadassah-Hebrew University Medical Center, Jerusalem, 91120, Israel. [55]Oxford NIHR Biomedical Research Centre, Oxford University Hospitals Trust, Headington, Oxford, OX3 7LE, UK. [56]Center for Genome Science, Korea National Institute of Health, Administration Complex, Chungcheongbuk-do, Republic of Korea. [57]Center of Biostatistics and Bioinformatics,

University of Mississippi Medical Center, Jackson, Mississippi, 39215, USA. [58]University of Exeter Medical School, University of Exeter, Exeter, EX2 5DW, UK. [59]Human Genetics Center, The University of Texas Graduate School of Biomedical Sciences at Houston, The University of Texas Health Science Center at Houston, Houston, Texas, 77030, USA. [60]Department of Endocrinology and Metabolism, Shanghai Diabetes Institute, Shanghai Jiao Tong University Affiliated Sixth People's Hospital, 600 Yishan Road, Shanghai, 200233, China. [61]Department of Statistics, Seoul National University, Shilim-dong, Kwanak-gu, Seoul, 151-742, Republic of Korea. [62]The Biostatistics Center, The George Washington University, Rockville, Maryland, 20852, USA. [63]Research, South Texas Veterans Health Care System, San Antonio, Texas, 78229, USA. [64]Department of Paediatrics, Yong Loo Lin School of Medicine, National University of Singapore, National University Health System, 119228, Singapore. [65]Division of Human Genetics, Genome Institute of Singapore, A*STAR, 138672, Singapore. [66]National Heart and Lung Institute, Cardiovascular Sciences, Hammersmith Campus, Imperial College London, London, W12 0NN, UK. [67]Faculty of Health Sciences, Institute of Clinical Medicine, Internal Medicine, University of Eastern Finland, Kuopio, Finland. [68]Kuopio University Hospital, Kuopio, Finland. [69]Interdisciplinary Program in Bioinformatics, Seoul National University, Seoul, 151-747, Republic of Korea. [70]Institute of Health Sciences, University of Oulu, Oulu, 90014, Finland. [71]Translational Laboratory in Genetic Medicine (TLGM), Agency for Science, Technology and Research (A*STAR) Singapore, 138648, Singapore. [72]General Medicine Division, Massachusetts General Hospital and Department of Medicine, Harvard Medical School, Boston, Massachusetts, 02114, USA. [73]Department of Genetics, University of North Carolina, CB 7264, Chapel Hill, North Carolina, 27599, USA. [74]Department of Biostatistics, University of Liverpool, Daulby Street, Liverpool, L69 3GA, UK. [75]Jackson Heart Study, University of Mississippi Medical Center, Jackson, Mississippi, USA. [76]Department of Human Genetics, McGill University, Stewart Biology Building, Room N5-13, Montreal, Quebec, H3A 1B1, Canada. [77]Research Institute of the McGill University Health Centre, Montreal, Quebec, H3H 2R9, Canada. [78]Department of Medicine, Division of Endocrinology, Diabetes and Nutrition, and Program in Personalized and Genomic Medicine, University of Maryland School of Medicine, Baltimore, Maryland, 21201, USA. [79]Department of Public Health and Primary Care, Institute of Public Health, University of Cambridge, Cambridge, CB1 8RN, UK. [80]Department of Laboratory Medicine & Institute for Human Genetics, University of California, San Francisco, San Francisco, California, 94143-0794, USA. [81]Blood Systems Research Institute, San Francisco, California, 94118, USA. [82]Division of Endocrinology and Metabolism, Department of Medicine, McGill University, Royal Victoria Hospital, Room A3.09, Montreal, Quebec, H3A 1A1, Canada. [83]Center for Vascular Prevention, Danube University Krems, Krems, 3500, Austria. [84]Cardiovascular & Metabolic Disorders Program, Duke-NUS Graduate Medical School Singapore, 169857, Singapore. [85]Department of Endocrinology and Metabolism, All India Institute of Medical Sciences, New Delhi, 110029, India. [86]Life Sciences Institute, National University of Singapore, 117456, Singapore. [87]Department of Statistics and Applied Probability, National University of Singapore, 117546, Singapore. [88]Department of Preventive Medicine, Keck School of Medicine, University of Southern California, Los Angeles, California, 90089-9073, USA. [89]Department of Physiology & Biophysics, Keck School of Medicine, University of Southern California, Los Angeles, California, 90089-9073, USA. [90]Diabetes and Obesity Research Institute, Keck School of Medicine, University of Southern California, Los Angeles, California, 90089-9073, USA. [91]College of Public Services, Jackson State University, Suite #701, Jackson, Mississippi, 39213, USA. [92]Department of Physiology and Biophysics, University of Mississippi Medical Center, Jackson, Mississippi, 39216, USA.
